# Dynamic decision-support for engineering course evaluation and improvement: a case study of the electric machinery and drives course

**DOI:** 10.1038/s41598-026-60717-1

**Published:** 2026-07-07

**Authors:** Chengling Lu, Yanxue Zhang

**Affiliations:** School of Electrical and Photoelectric Engineering, West Anhui University, Lu’An, 237012 China

**Keywords:** Engineering education, Dynamic teaching evaluation, Interval-valued intuitionistic fuzzy set, Entropy-weighted TOPSIS, Markov chain, Dynamic shortcoming repair coefficient, Electric machinery and drives, Engineering, Mathematics and computing

## Abstract

Engineering education accreditation emphasizes that course evaluation should enable continuous improvement. However, existing evaluation models are often constrained by static characteristics, single-dimensional assessment, insufficient predictive capability, and a disconnected closed-loop optimization mechanism. To address these challenges, this study develops a dynamic educational decision-support framework encompassing evaluation, diagnosis, prediction, and optimization. Using five-period data from 2021 to 2025 (n = 882) for the Electric Machinery and Drives (EMD) course, the framework is empirically demonstrated the proposed framework on a single-course longitudinal dataset, establishing 16 dynamic evaluation indicators covering both student and instructor dimensions. The framework integrates temporally adaptive weighting, correlation-aware multidimensional evaluation, trend-aware shortcoming diagnosis, and uncertainty-aware prediction. Specifically, sliding-window entropy weighting captures temporal changes in indicator importance, Mahalanobis-distance TOPSIS accounts for inter-indicator correlations, Markov prediction with Bootstrap confidence intervals estimates future state distributions, and D-SRC is formulated as a trend-aware extension of the Shortcoming Repair Coefficient to support proactive teaching intervention. On the basis of the three traditional dimensions (importance, deficiency, and student differentiation), the short-term evolutionary trend of indicator scores is introduced to realize bottleneck identification with both status diagnosis and prospective early warning. A three-dimensional, nine-measure optimization path is then formulated. The five-year analysis showed that the weights of technology-driven and ethics-related indicators changed substantially, with b10 and b15 increasing by 228% and 150%, respectively. M-TOPSIS provided correlation-aware evaluation and increased the SD-based relative dispersion of closeness coefficients by 10.7%, while Markov prediction with Bootstrap confidence intervals generated a short-term baseline projection of the 2026 excellent rate. After D-SRC-guided interventions for b15, b10, and b8, the corresponding scores increased by 7.7, 7.3, and 6.3 points, and the overall excellent rate increased from 24.1% to 39.6%. The proposed framework provides a decision-support tool for engineering education continuous improvement. By incorporating temporal trend information, D-SRC extends static shortcoming diagnosis toward prospective early warning and supports targeted intervention planning. The three-dimensional, nine-measure strategy further links diagnosis with teaching improvement, supporting the transition from static course assessment to dynamic, feedback-oriented educational decision support. Future studies should validate the framework across additional courses and institutions.

## Introduction

### Background: evolving evaluation demands driven by engineering education accreditation

The continuous advancement of engineering education accreditation places greater demands on the quality assurance of course instruction. International engineering accreditation standards, centered on the Washington Accord, emphasize the quality assurance concept of continuous improvement, which mandates that course evaluation not only objectively reflect the current instructional status but also possess the capability to diagnose issues and guide improvement^1,2^. Consequently, constructing a scientific, dynamic, and operational course evaluation system is relevant to course quality assurance and continuous improvement.

A course evaluation system should embody four fundamental characteristics: (1) dynamic adaptability, allowing evaluation priorities to be adjusted in response to technological advances and evolving industry demands. (2) multidimensionality, comprehensively considering instructor performance, student learning outcomes, and industry requirements. (3) predictive capability, enabling the anticipation of instructional outcome trends and providing forward-looking support for teaching decisions. (4) closed-loop optimization, translating diagnostic results into actionable improvement measures, thereby forming an "evaluation–diagnosis–improvement–re-evaluation" cycle^3–5^.

### Course characteristics: the specificity of the EMD course

The Electric Machinery and Drives course, a core course in the Electrical Engineering and Automation program, possesses three prominent characteristics:

- *Strong practicality*: The course includes numerous practical activities, such as motor structure analysis, electromagnetic parameter calculation, starting/braking/speed control system design, and fault diagnosis. Students are required to apply theoretical knowledge in real or simulated engineering scenarios. The evaluation system must capture indicators reflecting practical competence.
- *High interdisciplinary integration*: The course integrates knowledge from electromagnetics, mechanical dynamics, automatic control theory, and power electronics. Evaluation indicators should reflect students’ ability to comprehensively apply interdisciplinary knowledge.
- *Close industrial relevance*: Course content is closely aligned with national motor efficiency standards, industry standards, and technological updates such as permanent magnet synchronous motor (PMSM) drives and silicon carbide (SiC) device applications. The evaluation system must dynamically respond to technological advances and adjust evaluation priorities in a timely manner.

These characteristics indicate that the EMD course cannot rely solely on traditional static evaluation methods designed for theoretical courses; instead, a dynamic evaluation system that adapts to technological and industrial development is necessary.

### Problem statement: limitations of existing evaluation methods

Despite extensive research, existing evaluation methods still exhibit deficiencies in dynamic adaptability, multidimensional assessment, predictive capability, and closed-loop optimization:

- *Static characteristics*: Methods such as Analytic Hierarchy Process (AHP), fuzzy comprehensive evaluation, and entropy weighting generate fixed indicator weights, preventing tracking of technological advances and evolving industry requirements^6–8^.
- *Single-dimensional evaluation*: Most studies focus exclusively on student performance or teaching effectiveness, lacking a synergistic perspective encompassing both instructors and students^9,10^.
- *Insufficient predictive capability*: Existing methods are mostly post hoc analyses, offering limited ability to anticipate future trends^11,12^.
- *Broken closed-loop mechanism*: Evaluation results often fail to translate into actionable improvement measures, impeding effective teaching enhancement^13^.

These limitations prevent traditional evaluation models from meeting the dynamic “continuous improvement” requirements mandated by engineering education accreditation.

More specifically, these limitations can be summarized as four sequential gaps in engineering course evaluation. First, static weighting methods cannot reflect the temporal evolution of indicator importance under rapid technological change. Second, conventional Euclidean-distance-based evaluation methods ignore correlations among teaching indicators, which may distort multidimensional ranking results. Third, static shortcoming diagnosis can identify current weaknesses but cannot provide early warning for indicators with deteriorating trends. Fourth, point prediction alone cannot quantify uncertainty, limiting its usefulness for risk-aware teaching management. These gaps indicate that the key issue is not the isolated use of individual evaluation techniques, but the lack of a problem-oriented decision-support workflow that connects dynamic evaluation, diagnosis, prediction, and intervention.

### Literature review: applications and limitations of multi-criteria decision methods

Multi-criteria decision-making (MCDM) methods have been widely applied in educational evaluation. This section reviews the applications and limitations of four representative methods—AHP and its fuzzy extensions, entropy weighting, TOPSIS, and Markov chain prediction—in the context of course evaluation.

- *Analytic Hierarchy Process (AHP) and fuzzy extensions*: AHP has been widely used in course evaluation^7^. However, it relies on precise pairwise comparisons and struggles to capture uncertainty in expert judgment^14–16^. Interval-Valued Intuitionistic Fuzzy Sets (IVIF) represent membership, non-membership, and hesitation intervals, effectively quantifying support, opposition, and uncertainty^17,18^. Prior work has combined IVIF with AHP to form the IVIF–AHP weighting method, which can quantify expert hesitation^19^.
- Entropy-based weighting methods determine objective indicator weights according to the dispersion of data, eliminating the need for subjective expert input. Extensions of this approach have incorporated IVIF to better capture uncertainty in expert assessments. However, due to their fundamentally static formulation, these methods are limited in representing the temporal evolution of indicator importance over multiple time periods^20,21^.
- *TOPSIS and improved versions*: The Technique for Order Preference by Similarity to Ideal Solution (TOPSIS) ranks alternatives based on distances to ideal solutions. Traditional TOPSIS uses Euclidean distance, which assumes indicator independence. In teaching evaluation, indicators are often strongly correlated, making Euclidean distance unreliable. This study employs an improved TOPSIS based on Mahalanobis distance, which accounts for indicator covariance and more accurately reflects distances in multidimensional space^22–24^.
- *Markov chain prediction*: Markov chains have been applied to predict performance and student state evolution^16,17^, identifying stable and fluctuating states. However, they are typically limited to single indicators and rarely integrated with multidimensional evaluation. Recent studies attempt to combine Mahalanobis distance, reinforcement learning, and adaptive weighting to enable dynamic weighting and prediction^25,26^.

Beyond the classical MCDM methods discussed above, recent methodological advances have focused on improving robustness through hybrid frameworks, fuzzy modeling, and systematic evaluation of normalization techniques. For instance, comparative studies of MCDM methods have shown that the choice of normalization strategy and data representation format (crisp vs. fuzzy) can significantly influence ranking outcomes, highlighting the importance of carefully designed preprocessing and weighting procedures^27^. Similarly, hybrid MCDM frameworks combining fuzzy weighting methods with advanced ranking techniques have demonstrated improved stability and reliability in complex decision environments under uncertainty^28^. These developments indicate a clear trend toward integrated MCDM frameworks, where multiple complementary methods are combined to enhance decision robustness rather than relying on a single technique.

Beyond the education domain, recent studies in data-driven decision-support systems and benchmarking frameworks have demonstrated the applicability of multi-dimensional evaluation and optimization approaches across diverse fields. For example, a data-driven benchmarking framework for sustainability performance assessment has been proposed to enable gap analysis and targeted improvement strategies based on minimum performance thresholds rather than average scores^29^. Similarly, in the context of e-commerce adoption in agriculture, the Technology-Organization-Environment (TOE) framework has been integrated with social identity theory, demonstrating that multidimensional structural models can capture complex adoption behaviors through mediating mechanisms^30^. In the domain of finance, a multi-criteria decision-support approach has been applied to examine blockchain adoption, highlighting the importance of structured frameworks for addressing regulatory, technical, and compliance challenges^31^. These studies collectively indicate that the core methodological logic—integrating multi-criteria evaluation with decision-support and actionable improvement pathways—is not limited to a single domain but represents a general class of data-driven analytical frameworks.

Situated within this broader methodological landscape, the present study adapts these general decision-support principles to the specific context of engineering education continuous improvement. While the indicator system is domain-specific, the underlying framework structure follows a transferable decision-support logic that aligns with benchmarking and multi-criteria evaluation approaches documented in other disciplines.

### Research gap and positioning of this study

From the literature review, a clear research gap emerges: no integrated framework has been reported that can simultaneously handle expert uncertainty, dynamic evolution of indicators, multidimensional ranking, future trend prediction, and optimization path generation. Specifically:

- IVIF–AHP can address expert hesitation but produces static weights, failing to capture temporal evolution of indicator importance;
- Entropy weighting generates objective weights but remains static and detached from expert knowledge;
- TOPSIS provides ranking but ignores indicator correlation and lacks predictive capability;
- Markov chains predict trends but are often limited to single indicators and rarely integrated with multidimensional evaluation.

Integrating these methods into a dynamic course-evaluation workflow remains an unresolved methodological and practical issue. The current study aims to address this gap by proposing an integrated framework that will be detailed in the following sections.

To clarify the problem-driven rationale for method selection, Table 1 summarizes how each component addresses a specific educational evaluation challenge. The purpose of this design is not to stack multiple MCDM techniques, but to construct a sequential decision-support workflow in which each component addresses a distinct limitation of conventional course evaluation.

**Table 1:** Problem–method mapping of the dynamic educational decision-support framework.

Engineering education challenge	Limitation of conventional approach	Adopted strategy	Purpose
Indicator importance changes over time	Static weighting cannot capture temporal evolution	Sliding-window entropy weighting	Temporal adaptation
Teaching indicators are correlated	Euclidean-distance TOPSIS assumes indicator independence	Mahalanobis-distance TOPSIS	Correlation-aware evaluation
Current weaknesses may not reveal future risks	Static SRC is trend-blind	Trend-aware D-SRC	Prospective diagnosis
Forecasts require uncertainty information	Point prediction does not provide risk boundaries	Markov prediction with Bootstrap confidence intervals	Uncertainty-aware forecasting
Evaluation results need to guide improvement	Evaluation and intervention are often disconnected	Three-dimensional, nine-measure optimization path	Intervention-oriented feedback

Similar data-driven decision-support and benchmarking frameworks have been widely explored in other disciplines; however, their integration with dynamic weighting, correlation-aware evaluation, and trend-based diagnostic mechanisms in engineering education remains underexplored.

### Research contributions

The proposed framework is developed by a dynamic educational decision-support framework by adapting mature evaluation and prediction techniques to the context of engineering course continuous improvement. The main contributions are as follows.

1. Framework contribution: a structured decision-support workflow.
  This study constructs a framework that links dynamic weighting, correlation-aware evaluation, trend-aware diagnosis, uncertainty-aware prediction, and targeted intervention. The framework supports a transition from retrospective assessment toward a feedback-oriented decision-support process for engineering education continuous improvement.
2. Problem-oriented methodological adaptation.
  Mature MCDM and prediction methods are adapted to address specific challenges in course evaluation. Sliding-window entropy weighting captures temporal changes in indicator importance; Mahalanobis-distance TOPSIS accounts for correlations among teaching indicators; and Markov prediction with Bootstrap confidence intervals provides uncertainty-aware forecasting of teaching performance states.
3. Trend-aware shortcoming diagnosis using D-SRC.
  D-SRC is formulated as a trend-aware extension of the conventional SRC. It incorporates the temporal evolution of indicator scores into bottleneck diagnosis, thereby supporting proactive teaching intervention rather than only post hoc identification of current weaknesses.
4. Empirical demonstration using five-year EMD course data.
  Using five-period data from the Electric Machinery and Drives course from 2021 to 2025 (n = 882), the framework is empirically demonstrated through dynamic weight evolution, M-TOPSIS comparison, Markov prediction with confidence intervals, and targeted intervention validation.

## Construction of the dynamic evaluation and prediction model

### Construction of the dynamic indicator system

#### Evolution logic of the indicator system

The construction of the evaluation indicator system is a fundamental step in course evaluation, as its scientific rigor and completeness directly affect the reliability of results. This study establishes a 16-indicator dynamic evaluation system covering both student and instructor dimensions, aligned with new engineering education accreditation requirements and teaching practice development. All 16 indicators have been continuously tracked from 2021 to 2025, enabling complete five-year time-series analysis.

The core design principles of the indicator system include two aspects:

- Accelerated technological iteration: Rapid developments in permanent magnet synchronous motor (PMSM) drives, direct torque control (DTC), and silicon carbide (SiC) device applications require the curriculum to enhance technological frontier adaptability. Accordingly, Technological Frontier Adaptability (b7) is designed within the student dimension (P4) to reflect students’ alignment with emerging motor control technologies.
- Normalization of hybrid teaching: In the post-pandemic era, the use of virtual simulation platforms (e.g., MATLAB/Simulink, Ansys Maxwell) and VR/AR technologies in teaching has become widespread. Hence, Virtual Simulation Teaching Effectiveness (b16) is designed within the instructor dimension (P9) to capture the effectiveness of simulation-based and virtual laboratory instruction.

For clarity, the 16 indicators are categorized into the student dimension (Table 2) and the instructor dimension (Table 3), comprising four primary indicators (P1–P4) and five primary indicators (P5–P9), respectively, with associated secondary indicators.

**Table 2:** Student dimension indicators.

Primary indicator	Secondary indicator	Definition/quantification	Data source
Practical motor ability (P1)	Motor starting/braking debugging (b1)	Accuracy of star-delta starting configuration, soft starter parameter setting, braking resistor selection	Lab reports / simulation results
	Motor speed control implementation (b2)	Compliance of V/F control, vector control, or DTC parameter tuning; effectiveness of speed regulation	Design drawings / drive logs
Technical documentation ability (P2)	Motor selection and design calculation standards (b3)	Completeness of electromagnetic torque calculation, thermal load verification, efficiency estimation	Course assignments / industry mentor evaluation
	Motor efficiency test reports (b4)	Compliance with GB 18,613 / IE3/IE4 efficiency standards, accuracy of loss analysis	Project reports
Collaborative innovation ability (P3)	Motor-load matching collaboration (b5)	Team task contribution, completeness of matching analysis for fan/pump/conveyor applications	Collaboration platform data
	Motor drive system integration (b6)	Completeness of PLC + inverter + HMI integrated debugging, proficiency in communication protocol configuration	Training platform records
Technological frontier adaptability (P4)	Technological frontier adaptability (b7)	Alignment of course content with PMSM, DTC, SiC device, and industrial robot servo technologies	Syllabus review / expert panel

**Table 3:** Instructor dimension indicators.

Primary indicator	Secondary indicator	Definition/quantification	Data source
Teaching competence (P5)	Industrial motor application case updates (b8)	Proportion of annual real engineering cases (e.g., EV drives, industrial robots), penetration of latest industry standards	Syllabus / lecture materials
	On-site teaching ability (b9)	Frequency of motor disassembly/fault simulation site teaching, completeness of equipment demonstration	Teaching logs / student feedback
Integration of technological frontiers (P6)	Modern motor control technology (b10)	Proportion of course hours covering vector control, DTC, sensorless control; depth of algorithm understanding	Lesson plan review
	Motor efficiency standards and energy-saving technology (b11)	Integration of IE3/IE4/IE5 efficiency standards, copper rotor technology, high-efficiency motor design principles	Course project analysis
Teaching process execution (P7)	Motor virtual simulation experiments (b12)	Frequency of MATLAB/Simulink or Ansys Maxwell simulation experiments, safety compliance of physical equipment operations	Lab records / monitoring videos
	Motor safety standards penetration (b13)	Coverage of high-voltage safety, insulation testing, thermal protection, mechanical hazard prevention	Assessment results
Industry–education integration depth (P8)	Motor manufacturer/drive enterprise engineer participation (b14)	Teaching hours of industry experts, number of projects converted into practice	Enterprise teaching records
	Engineering ethics and safety responsibility (b15)	Intensity of safety awareness training, assessment of professional responsibility (consequences of motor failure)	Ethics tests / behavioral observations
Virtual simulation teaching effectiveness (P9)	Virtual simulation teaching effectiveness (b16)	Completion rate of virtual motor disassembly/assembly, accuracy of electromagnetic field simulation operations, success rate of VR training tasks	Simulation platform logs

#### Characteristics and advantages of the indicator system

The proposed indicator system exhibits three prominent features:

- Dual-dimension synergy: By categorizing indicators into student and instructor dimensions, the system allows analysis of the synergy between teaching input and student competence enhancement. As demonstrated in Section "Dual-dimension mechanism verification", the synergy coefficient quantifies alignment between instructor effort and student performance.
- Dynamic adaptability: Indicators such as Technological Frontier Adaptability (b7) and Virtual Simulation Teaching Effectiveness (b16) are inherently dynamic, with importance evolving alongside technological advancement and teaching modes. The dynamic weight computation captures these changes, ensuring the evaluation system remains up to date.
- Operability: Each indicator has clearly defined quantification methods and data sources, ensuring repeatability and comparability. Data sources include lab reports, design drawings, simulation platform logs, drive logs, syllabus reviews, and expert panel scoring, reflecting a multi-source data integration approach tailored to motor and drive education.

Importantly, the proposed framework is not a collection of independent methods but a sequential decision-support pipeline in which each module is activated to address a specific structural limitation of conventional educational evaluation systems.

### Dynamic IVIF-entropy weighting procedure

Once the indicator system is constructed, determining the weight of each indicator becomes critical. Traditional methods rely either on expert experience (subjective weighting) or data distribution (objective weighting), each with advantages and limitations. In this study, IVIF-based subjective weighting and entropy-based objective weighting are combined through a dynamic weighting procedure. The purpose is to allow indicator weights to reflect both expert judgment and temporal changes in data dispersion.

#### Sliding window entropy weight method: dynamic weight evolution

Traditional entropy weighting computes all historical data at once, assuming constant indicator importance. In engineering education, indicator importance naturally evolves over time due to technological and industrial changes.

A sliding window strategy is adopted: the weight in year t is calculated based on data from the most recent T years rather than all historical data. This captures medium-term trends while smoothing out annual fluctuations.

The choice of window width is critical. Small windows (e.g., *T* = 2) respond quickly but are sensitive to anomalies; large windows (e.g., *T* = 5) provide greater smoothness. Given that all 16 indicators have complete five-year panel data from 2021 to 2025, this study adopts a 5-year sliding window [*t*-4, *t*], which utilizes the full temporal span of the dataset and provides the most stable weight estimation. The value *T* = 5 was therefore selected as the baseline window because the dataset contains five complete annual cohorts, and the purpose of this study is to capture medium-term evolution rather than short-term annual fluctuations. Shorter windows may improve responsiveness but are more sensitive to single-year anomalies. Additional checks using shorter windows produced the same set of high-priority shortcoming indicators, indicating that the main conclusions were not driven solely by the choice of T = 5. This modification does not change the fundamental principle of entropy weighting while adapting the calculation to the longitudinal structure of the five-year dataset.

The mathematical formulation is as follows: let the evaluation matrix for year *t* be ${\mathbf{X}}^{(t)} = [x_{ij}^{(t)} ]_{n \times m}$, where n is the number of samples and m = 16 is the number of indicators. The information entropy of the *j*-th indicator over the window [*t*-4, *t*] is:

1 $$e_{j}^{(t)} = - \frac{1}{\ln (5n)}\sum\limits_{\tau = t - 4}^{t} {\sum\limits_{i = 1}^{n} {p_{ij}^{(\tau )} } } \ln p_{ij}^{(\tau )}$$

where

2 $$p_{ij}^{(\tau )} = \frac{{x_{ij}^{(\tau )} }}{{\sum\nolimits_{\tau = t - 4}^{t} {\sum\nolimits_{i = 1}^{n} {x_{ij}^{(\tau )} } } }}$$

where $x_{ij}^{(\tau )}$ denotes the observed value of indicator *j* for sample *i* in year *τ*; $p_{ij}^{(\tau )}$ represents the normalized proportion of indicator *j* at sample level within the five-year sliding window [*t* − 4, *t*].

The denominator is ln(5*n*) to normalize for the 5-year window. The objective entropy weight is then:

3 $$w_{j}^{(t)} = \frac{{1 - e_{j}^{(t)} }}{{\sum\nolimits_{k = 1}^{m} {(1 - e_{k}^{(t)} )} }},\quad j = 1,2, \ldots ,m$$

It should be emphasized that in Eq. (2), the denominator is set to ln(5n) rather than the conventional ln(n) used in classical entropy-weighting methods. This adjustment reflects the effective sample size being expanded fivefold by the five-year sliding window, thereby ensuring consistency in the normalization of information entropy. This treatment is fully consistent with the fundamental principle of classical entropy weighting based on ln(n), while being adapted to accommodate the enlarged window.

**Fig. 1:**
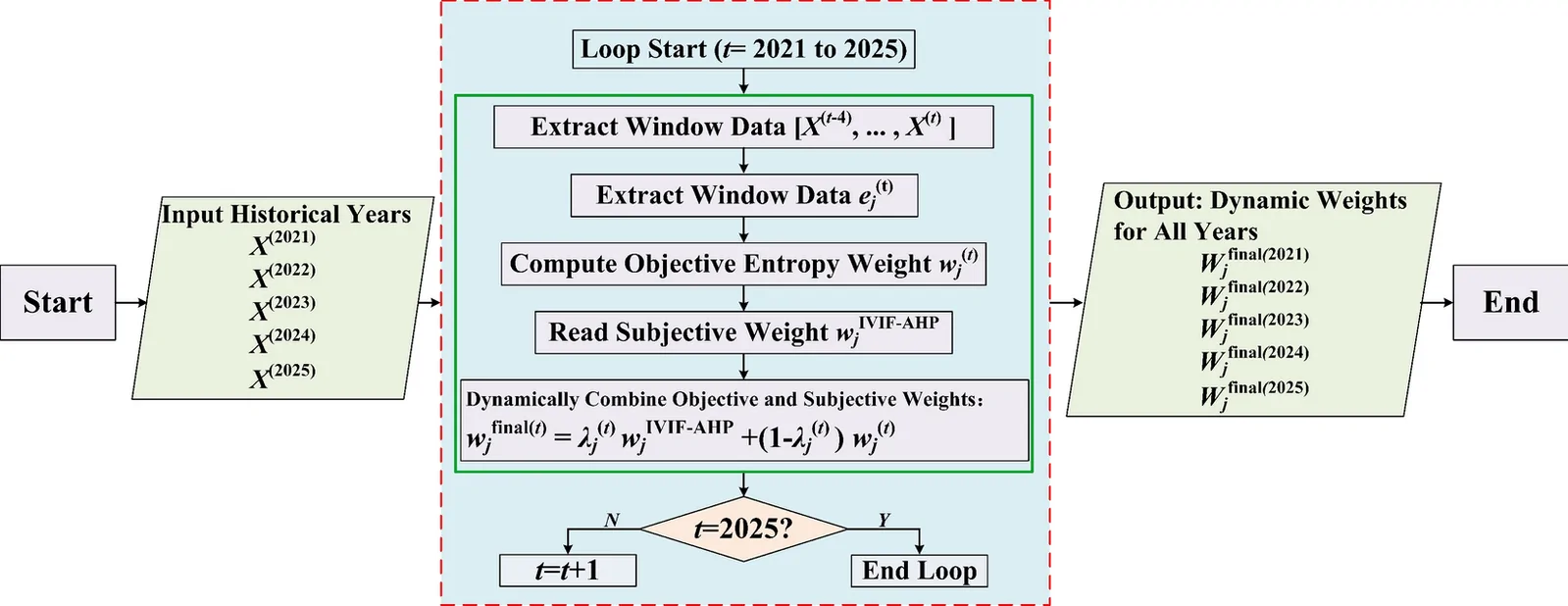
Workflow of the sliding-window entropy-weight method.

The computational procedure of the sliding-window entropy-weight method is illustrated in Fig. 1.

#### Dynamic fusion of subjective and objective weights

The sliding-window entropy-weighting method provides objective weights $w_{j}^{(t)}$, which are entirely based on the data distribution and reflect the importance of indicators at the data level. However, relying solely on objective weights may overlook expert knowledge, especially when data quality is low or sample sizes are limited, which can lead to instability or deviation from actual importance.

Subjective weights $w_{j}^{{\text{IVIF - AHP}}}$ are derived from the IVIF-AHP method, which quantifies expert hesitation through pairwise comparisons based on interval-valued intuitionistic fuzzy sets^19^. These weights capture the consensus assessment of indicator importance. Unlike objective weights, subjective weights are static and represent the shared judgment of the expert group.

The final weight for the t-th year is defined as a convex combination of subjective and objective weights:

4 $$w_{j}^{{{\mathrm{final}}(t)}} = \lambda_{j}^{(t)} \cdot w_{j}^{{\text{IVIF - AHP}}} + (1 - \lambda_{j}^{(t)} ) \cdot w_{j}^{(t)}$$

The core design lies in determining the fusion coefficient $\lambda_{j}^{(t)} \in [0,1]$.$\lambda_{j}^{(t)}$ is not a fixed constant but dynamically adjusted according to the situation, reflecting an organic integration of “expert knowledge” and "data-driven" information. Specifically, $\lambda_{j}^{(t)}$ is determined by two factors:

- Expert hesitation $\pi_{j}$: Derived from the IVIF-AHP computation,$\pi_{j}$ indicates the level of consensus among experts regarding indicator *j*. Larger $\pi_{j}$ signifies higher disagreement, which reduces the reliability of subjective weights, so a smaller value of $\lambda_{j}^{(t)}$ should be adopted.
- Data dispersion $CV_{j}^{(t)}$: The coefficient of variation of indicator *j* within the window [*t*-4, *t*], reflecting the diversity of student performance. Larger $CV_{j}^{(t)}$ indicates richer information, which improves the reliability of objective weights, so a larger weight proportion should be assigned to the objective component.

The specific rules for $\lambda_{j}^{(t)}$ follow those in Eq. (19) of^19^ and are not repeated here. This design allows the fusion coefficient to respond to both data distribution and expert consensus: when expert opinions are highly consistent ($\pi_{j}$ small) and data dispersion is low ($CV_{j}^{(t)}$ small), subjective weights dominate; when expert disagreement is high ($\pi_{j}$ large) and data dispersion is high ($CV_{j}^{(t)}$ large), objective weights dominate.

Through this dynamic fusion mechanism, the complementary advantages of “expert knowledge” and "data-driven" approaches are realized. For example, when an indicator’s weight changes by over 200% over five years (as will be seen for indicator b10), relying solely on static expert judgment cannot capture this evolution, while using only the entropy-weight method may be affected by single-year anomalies. The dynamic fusion smooths fluctuations via the sliding window while preserving the expert baseline, achieving an optimal balance between smoothness and sensitivity.

### Improved TOPSIS: incorporating Mahalanobis distance

After obtaining the dynamic weights of the indicators, the next step is to perform a comprehensive evaluation and ranking of teaching performance based on these weights. TOPSIS is widely adopted due to its intuitive geometric interpretation and computational simplicity. However, the conventional TOPSIS has a fundamental limitation: it measures the closeness to the ideal solution using Euclidean distance, which implicitly assumes independence among the indicators.

#### Motivation for Mahalanobis distance

In teaching evaluation, indicators are often strongly correlated. For example:

- "Modern Motor Control Technology" (b10) is highly positively correlated with "Technological Frontier Adaptability" (b7), as the former is a key component of the latter.
- "Motor Virtual Simulation Experiments" (b12) is highly positively correlated with "Virtual Simulation Teaching Effectiveness" (b16), as both reflect the integration of simulation tools in teaching.

Euclidean distance ignores such correlations and treats each indicator as an independent dimension, which mathematically assumes an isotropic indicator space. When correlations exist, the actual sample distribution is ellipsoidal rather than spherical. Euclidean distance underestimates some directional differences and overestimates others, causing distortion in distance measurement^26^.

Mahalanobis distance is introduced to address this issue. By incorporating the covariance matrix, it accounts for inter-indicator correlation structures and more accurately reflects the true distances of samples in multidimensional space^32^. Geometrically, it can be interpreted as whitening the original data to remove correlations before calculating Euclidean distances.

Table 4 compares commonly used distance metrics in TOPSIS and highlights the advantages of Mahalanobis distance.

**Table 4:** Comparison of different distance metrics in TOPSIS.

Distance metric	Formula	Advantages	Limitations
Euclidean distance	$\sqrt {\sum {(x_{i} - y_{i} )^{2} } }$	Intuitive, simple, low computation	Assumes independent indicators; ignores correlation
Manhattan distance	$\sum | x_{i} - y_{i} |$	Robust to outliers	Also ignores indicator correlations, resulting in substantial information loss
Chebyshev distance	$\max |x_{i} - y_{i} |$	Focuses on the maximum deviation, suitable for extreme-sensitive scenarios	Neglects information from most indicators, causing severe information loss
Mahalanobis distance	$\sqrt {({\mathbf{x}} - {\mathbf{y}})^{T} \Sigma^{ - 1} ({\mathbf{x}} - {\mathbf{y}})}$	Accounts for correlations, scale-invariant, automatically eliminates the influence of dimensions	Covariance matrix estimation required, computationally intensive

Based on the correlations among teaching evaluation indicators, this study adopts Mahalanobis distance within the TOPSIS procedure to obtain a correlation-aware closeness measure, referred to as M-TOPSIS. Therefore, the use of Mahalanobis distance is a context-aware adaptation for correlated teaching indicators.

#### Steps for the improved TOPSIS computation

**Fig. 2:**
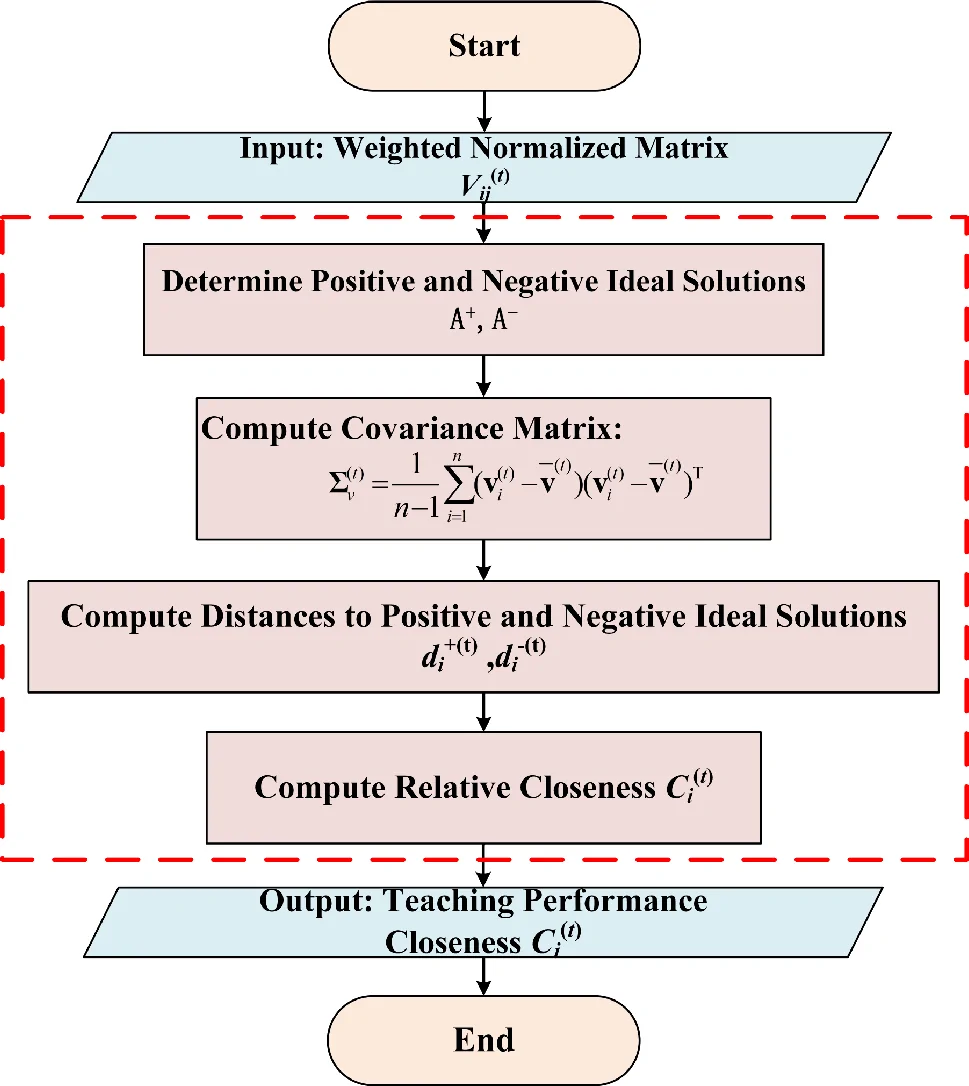
Steps for the improved TOPSIS computation.

The computation procedure for the improved TOPSIS is illustrated in Fig. 2 and proceeds as follows:

**Step 1:** Construct the weighted normalized matrix.

The original evaluation data is first normalized to a dimensionless form and then multiplied by the dynamic weights.

5 $$v_{ij}^{(t)} = w_{j}^{{{\mathrm{final}}(t)}} \cdot \frac{{x_{ij}^{(t)} }}{{\sqrt {\sum\limits_{i = 1}^{n} {(x_{ij}^{(t)} )^{2} } } }},\quad i = 1, \ldots ,n;\quad j = 1, \ldots ,m$$

**Step 2:** Determine the positive and negative ideal solutions.

The positive ideal solution consists of the maximum values of each indicator, and the negative ideal solution consists of the minimum values of each indicator, as shown in Eqs. (6) and (7).

6 $${\mathbf{A}}^{ + } = (\max_{i} v_{i1}^{(t)} ,\max_{i} v_{i2}^{(t)} , \ldots ,\max_{i} v_{im}^{(t)} )$$

7 $${\mathbf{A}}^{ - } = (\min_{i} v_{i1}^{(t)} ,\min_{i} v_{i2}^{(t)} , \ldots ,\min_{i} v_{im}^{(t)} )$$

**Step 3:** Compute the covariance matrix.

The covariance matrix characterizes linear relationships between indicators:

8 $${{\boldsymbol{\Sigma}}}_{v}^{(t)} = \frac{1}{n - 1}\sum\limits_{i = 1}^{n} {({\mathbf{v}}_{i}^{(t)} - \overline{{\mathbf{v}}}^{(t)} )} ({\mathbf{v}}_{i}^{(t)} - \overline{{\mathbf{v}}}^{(t)} )^{{\mathrm{T}}}$$

where $\overline{{\mathbf{v}}}^{(t)}$ denotes the mean vector of all samples in year *t*.

**Step 4:** Compute Mahalanobis distances.

Using the inverse of the covariance matrix, calculate the Mahalanobis distance of each sample to the positive and negative ideal solutions:

9 $$d_{i}^{ + (t)} = \sqrt {({\mathbf{v}}_{i}^{(t)} - {\mathbf{A}}^{ + } )^{{\mathrm{T}}} ({{\boldsymbol{\Sigma}}}_{v}^{(t)} )^{ - 1} ({\mathbf{v}}_{i}^{(t)} - {\mathbf{A}}^{ + } )}$$

10 $$d_{i}^{ - (t)} = \sqrt {({\mathbf{v}}_{i}^{(t)} - {\mathbf{A}}^{ - } )^{{\mathrm{T}}} ({{\boldsymbol{\Sigma}}}_{v}^{(t)} )^{ - 1} ({\mathbf{v}}_{i}^{(t)} - {\mathbf{A}}^{ - } )}$$

**Step 5:** Compute the relative closeness coefficient.

The relative closeness to the ideal solution is defined as:

11 $$C_{i}^{(t)} = \frac{{d_{i}^{ - (t)} }}{{d_{i}^{ + (t)} + d_{i}^{ - (t)} }},\quad i = 1, \ldots ,n$$

where $C_{i}^{(t)} \in [0,1]$ represents how close sample i is to the positive ideal solution, with larger values indicating better teaching performance.

It should be noted that Mahalanobis distances do not strictly preserve non-negative monotonicity as Euclidean distances do; however, in this study, they are used solely to measure relative proximity to the ideal solution. The closeness coefficient formula remains consistent with conventional TOPSIS for comparability. The empirical analysis presented later examines whether this approach increases the SD-based relative differentiation of teaching performance scores in this case study.

### Markov prediction model

The previous steps implement dynamic evaluation of teaching performance, yet such evaluation is inherently retrospective. To satisfy the continuous improvement requirement of engineering education accreditation, forward-looking predictive capability is also needed. Markov chains provide a simple and interpretable way to describe transitions among teaching performance states based on historical observations. In this study, the first-order Markov model is used as a transparent baseline forecasting approximation for short-term state evolution, rather than a complete causal model of educational outcomes. We acknowledge that educational outcomes are influenced by multiple cumulative and contextual factors, including curriculum reforms, instructor changes, cohort quality, teaching resources, and policy adjustments, which are not explicitly modeled in the current framework. Therefore, the Markov component is intended to provide an interpretable trend reference with uncertainty bounds, rather than a deterministic or exhaustive prediction of future course performance.

#### Application basis of Markov chains in educational prediction

A Markov chain is a stochastic process describing state transitions, whose core assumption is memorylessness: the future state depends only on the current state and is independent of earlier states. For educational prediction, this assumption is necessarily a simplification because course performance may reflect accumulated effects from previous reforms, instructor experience, student cohort characteristics, and external policy conditions. In this study, the first-order Markov assumption is therefore not treated as a complete representation of educational dynamics, but as a tractable approximation for short-term state projection under limited annual observations.

This choice is motivated by three practical considerations. First, the state-based form of the Markov model is transparent and easy to interpret for teaching management, because it describes transitions among educational performance levels such as Excellent, Good, Medium, Pass, and Fail. Second, given the five-year observation window, more complex higher-order Markov models or machine-learning time-series models would require more longitudinal data and may increase the risk of overfitting. Third, when combined with Bootstrap confidence intervals, the Markov component can provide an uncertainty-aware baseline projection rather than a single deterministic forecast.

Markov chains have been widely applied in educational prediction. For example, one study combined Markov chains with machine learning models to construct a hybrid ensemble predicting student engagement and academic performance in virtual learning environments, achieving 91.9% accuracy^33^. Their study revealed an important pattern: students in a high-engagement state tend to remain stable, students in low-engagement states struggle to improve without support, and students in medium-engagement states exhibit the most variability. This finding provides a theoretical basis for adaptive interventions.

Bayesian state-space models have also been used to dynamically track student risk evolution, capturing fluctuations more accurately than static logistic regression^34^. Building upon these insights, this study combines Markov chains with TOPSIS closeness coefficients to achieve multidimensional prediction of teaching performance.

#### State definition

Continuous closeness coefficients $C_{i}^{(t)}$ are discretized into a finite set of states. To improve interpretability for teaching management, continuous closeness coefficients are discretized into five states corresponding to the commonly used five-level educational performance standard, as shown in Table 5.

**Table 5:** State classification for teaching performance.

State	Closeness range	Grade	Corresponding score
S1	[0.8, 1.0]	Excellent	95
S2	[0.6, 0.8)	Good	85
S3	[0.4, 0.6)	Medium	75
S4	[0.2, 0.4)	Pass	60
S5	[0, 0.2)	Fail	30

The five-state discretization was adopted because it corresponds to the commonly used five-level educational performance categories: Excellent, Good, Medium, Pass, and Fail. The thresholds 0.8,0.6,0.4, and 0.2 provide an interpretable mapping between continuous TOPSIS closeness values and teaching-management categories. This setting balances interpretability and granularity: fewer states would provide overly coarse diagnostic information, whereas more states would be difficult to estimate reliably with five annual cohorts. This classification has clear implications for engineering education: S1 indicates full compliance with industry standards, S2 satisfies core requirements, S3 requires partial improvement, S4 meets only minimal teaching requirements, and S5 fails to meet educational quality standards.

#### Transition matrix and prediction algorithm

Based on frequency counts of historical state transitions, the state transition probability matrix can be constructed as

12 $$p_{kl} = \frac{{n_{kl} }}{{\sum\nolimits_{l = 1}^{5} {n_{kl} } }},\quad k,l = 1,2, \ldots ,5$$

Here,$n_{kl}$ denotes the number of samples transitioning from state *k* to state l. Each row of ${\mathbf{P}}$ sums to 1: $\sum\limits_{l = 1}^{5} {p_{kl} } = 1$_._

Let the state probability distribution at year (t) be the row vector ${{\boldsymbol{\uppi}}}^{(t)} = [\pi_{1}^{(t)} ,\pi_{2}^{(t)} , \ldots ,\pi_{5}^{(t)} ]$. The predicted distribution for year (*t* + 1) is

13 $${{\boldsymbol{\uppi}}}^{(t + 1)} = {{\boldsymbol{\uppi}}}^{(t)} \cdot {\mathbf{P}}$$

and for year *t* + *k*:

14 $${{\boldsymbol{\uppi}}}^{(t + k)} = {{\boldsymbol{\uppi}}}^{(t)} \cdot {\mathbf{P}}^{k}$$

**Fig. 3:**
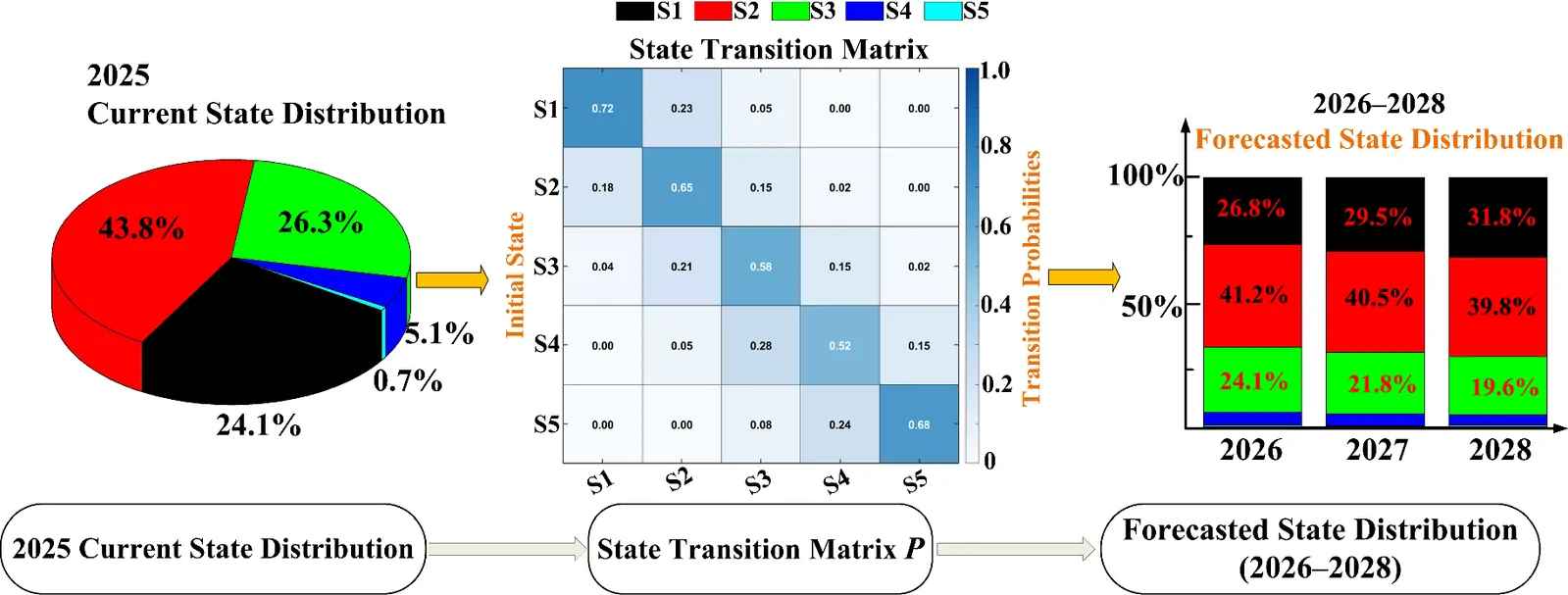
Schematic diagram of Markov chain forecast.

Using the actual 2025 state distribution and the transition matrix, Eqs. (13)–(14) are applied to predict teaching performance trends for 2026–2028. As illustrated in Fig. 3, without additional interventions, the course excellent rate (S1) is projected to increase from 24.1% in 2025 to 31.8% in 2028, the good rate (S2) remains around 40%, and the proportion of medium and lower states gradually decreases, indicating a continuous improvement trend.

#### Bootstrap confidence interval estimation

Point predictions are intuitive but do not reflect predictive uncertainty. To enhance reliability, the Bootstrap method is applied to resample the transition matrix and estimate confidence intervals.

Bootstrap is a resampling-based statistical inference technique^35^. The steps are as follows:

1. Perform (B = 1000) resamples with replacement on the original transition frequency matrix, generating a set of Bootstrap transition matrices $\{ {\mathbf{P}}^{(b)} \}_{b = 1}^{B}$. Each resample randomly selects the same number of samples as the original dataset, recalculates transition frequencies, and computes transition probabilities.
2. Apply Eqs. (13)–(14) to each ${\mathbf{P}}^{(b)}$ to obtain Bootstrap predicted distributions ${{\boldsymbol{\uppi}}}^{(t + k,b)}$.
3. Using the percentile method, take the 2.5% and 97.5% quantiles of the 1000 resamples to construct 95% confidence intervals for each state:
  15 $$[\pi_{l}^{(t + k,2.5\% )} ,\pi_{l}^{(t + k,97.5\% )} ],\quad l = 1, \ldots ,5$$

The Bootstrap method estimates prediction uncertainty and provides additional uncertainty information for educational decision-making. For example, the predicted excellent rate for 2026 is 26.8% (95% CI: 24.1%–29.5%), meaning there is 95% confidence that the true rate falls within this interval. This provides additional information beyond point estimates.

#### Reliability validation of the Markov chain

The prediction model is validated in three aspects: state classification sensitivity analysis, Markovian assumption testing, and rolling forecast error verification.

1. State classification sensitivity analysis
  Adjusting state thresholds may affect the stability of the transition matrix and prediction results. Thresholds were varied by ± 5% to verify robustness, and the resulting predictions were observed in Table 6.

**Table 6:** Sensitivity analysis of state classification.

State threshold adjustment	Predicted excellent rate 2026	Variation	Significant?
Baseline (Table 5)	26.8%	-	-
Lowered S1 threshold to 0.75	28.4%	+ 1.6%	No
Raised S1 threshold to 0.85	25.3%	-1.5%	No
S2 threshold adjusted simultaneously	27.2%	+ 0.4%	No
  Even when the S1 threshold is adjusted from 0.8 to 0.75 or 0.85, the predicted changes (1.6% and 1.5%) are smaller than the half-width of the Bootstrap confidence interval (~ 3.5%), indicating that the prediction results were not materially affected by small threshold perturbations. Therefore, the Markov prediction results are not materially affected by small perturbations in the state thresholds.
2. Markovian assumption testing
  The first-order Markov assumption was tested using a chi-square test:
  16 $$\chi^{2} = \sum\limits_{i,j,k} {\frac{{(n_{ijk} - \hat{n}_{ijk} )^{2} }}{{\hat{n}_{ijk} }}} \sim \chi_{{(s - 1)^{2} (s - 2)}}^{2}$$
  With s = 5 states, $\chi^{2} = 86.4 > \chi_{0.05}^{2} (48) = 65.2$, *p* < 0.05, rejecting the null hypothesis. This indicates potential higher-order dependencies in student performance evolution, consistent with cumulative learning characteristics. Although the chi-square test rejects the strict first-order Markov assumption, the Markov transition matrix is still employed as a first-order approximation of short-term state evolution rather than as a strictly validated stochastic generative model. This allows the model to capture coarse-grained transition tendencies in student performance states, providing interpretable probabilistic guidance for trend-aware prediction.
3. Rolling forecast error verification
  A rolling forecast approach was used to validate the predictive accuracy of the Markov model. For the 2024 prediction, a 3-year sliding window (2021–2023 data) was employed due to data availability; for the 2025 prediction, the full 5-year sliding window (2021–2024 data) was used, consistent with the main methodology. The predictions were compared with actual outcomes, as shown in Table 7.

**Table 7:** Rolling forecast error verification.

Year	Actual excellent rate	Predicted excellent rate	Absolute error	Relative error
2024	18.9%	19.8%	0.9%	4.8%
2025	24.1%	23.5%	0.6%	2.5%
Average	–	–	0.75%	3.65%

The mean absolute error was 0.75 percentage points, and the mean relative error was 3.65%, suggesting acceptable short-term predictive consistency in this case study. Despite potential higher-order dependencies, the first-order Markov approximation still provided a usable short-term baseline in this case study. Therefore, the Markov component is used primarily to provide a transparent baseline forecast with uncertainty intervals, while its limitations under higher-order dependence are explicitly acknowledged.

### Dynamic shortcoming repair coefficient (D-SRC)

The preceding sections provide dynamic weighting, correlation-aware evaluation, and uncertainty-aware prediction. However, these procedures do not by themselves determine which indicators should receive priority in teaching intervention. To support diagnosis-guided resource allocation, this study extends the conventional Shortcoming Repair Coefficient (SRC) by incorporating temporal trend information, leading to the Dynamic Shortcoming Repair Coefficient (D-SRC). This extension enables a shift from static diagnostic ranking to a trend-aware decision-support mechanism for prioritizing instructional intervention.

#### Foundational definition of the static SRC

The Shortcoming Repair Coefficient (SRC) is designed based on the bottleneck effect in systems theory, which states that the overall performance of a system is constrained by its weakest components. Unlike a simple “minimum value” approach, SRC simultaneously incorporates three dimensions:

1. Importance Dimension ($w_{j}^{{{\mathrm{final}}(t)}}$): Higher weights indicate greater significance of the indicator in teaching performance evaluation and a stronger bottleneck effect.
2. Deficiency Dimension $(1 - \overline{C}_{j}^{(t)} )$: Let $\overline{C}_{j}^{(t)}$ denote the average of the closeness coefficients $C_{i}^{(t)}$ for indicator* j* in year *t*. Poorer performance reflects greater improvement potential, warranting higher priority for intervention.
3. Student Differentiation Dimension $\sigma_{j}^{(t)} /\overline{x}_{j}^{(t)}$: Greater variability among students indicates instability in instructional effectiveness and potential challenges in teaching method adaptation.

Indicators with high values in all three dimensions represent critical shortcomings that are important, weak, and highly variable, and should be prioritized for remediation. The SRC is mathematically defined as:

17 $$SRC_{j}^{(t)} = w_{j}^{{{\mathrm{final}}(t)}} \cdot (1 - \overline{C}_{j}^{(t)} ) \cdot \frac{{\sigma_{j}^{(t)} }}{{\overline{x}_{j}^{(t)} }}$$

The SRC ranges from [0, + ∞); higher values indicate higher repair priority.

#### From static to dynamic: incorporation of the trend dimension

The conventional SRC formulation effectively identifies current teaching bottlenecks but remains inherently static, as it reflects only single-period performance. In educational practice, however, instructional improvement exhibits temporal dynamics. An indicator with a low current score but an improving trajectory suggests effective intervention and reduced urgency, whereas an indicator with acceptable performance but persistent deterioration may indicate an emerging future bottleneck requiring early warning.

To incorporate temporal sensitivity into diagnostic evaluation, the Static SRC is extended by introducing a trend-aware modulation component, resulting in the Dynamic Shortcoming Repair Coefficient (D-SRC):

18 $$DSRC_{j}^{(t)} = w_{j}^{{{\mathrm{final}}(t)}} \cdot (1 - \overline{C}_{j}^{(t)} ) \cdot \frac{{\sigma_{j}^{(t)} }}{{\overline{x}_{j}^{(t)} }} \cdot (1 + \alpha \cdot Trend_{j}^{(t)} )$$

where the additional term introduces temporal modulation based on recent historical evolution. Let $Trend_{j}^{(t)}$ denote the normalized temporal trend of indicator *j* over the observation window [*t* − 4, *t*], defined as:

19 $$Trend_{j}^{(t)} = \frac{{slope_{j}^{(t)} }}{max(|slope|)}$$

where slope $slope_{j}^{(t)}$ is obtained via simple linear regression, using year as the independent variable and the indicator mean as the dependent variable. The normalization term denotes the maximum absolute slope across all indicators, ensuring that $Trend_{j}^{(t)}$ ∈ [− 1,1] for cross-indicator comparability.

To ensure interpretability and avoid dominance over static diagnostic components, the modulation coefficient α is introduced. In this study, *α* = 0.2 is adopted as a balanced setting. A sensitivity analysis over *α* ∈ {0, 0.1, 0.2, 0.3, 0.5} confirms that the set of top three critical indicators (b15, b10, b8) remains unchanged across all tested values, indicating that the D-SRC ranking is robust to moderate variations in the trend adjustment coefficient.

To further validate the robustness of trend directionality under minimal sample size (five annual observations), a non-parametric Mann–Kendall trend test is employed as a supplementary check. The results confirm consistency between the linear trend direction and non-parametric trend inference for the key indicators.

In the final formulation, D-SRC integrates four complementary dimensions: indicator importance, performance deficiency, inter-sample differentiation, and temporal evolution. The trend component serves as a modulation factor rather than a standalone decision driver, ensuring that intervention prioritization is jointly determined by multidimensional evaluation rather than temporal variation alone.

#### Diagnostic logic and interpretation of D-SRC

Higher D-SRC values indicate higher repair priority. Compared with static SRC, D-SRC introduces temporal information into shortcoming diagnosis and enables the framework to consider not only the current status of an indicator but also its recent evolutionary tendency. It should be emphasized that D-SRC is a composite diagnostic coefficient. The trend term does not independently determine the repair priority; rather, it modulates the static SRC together with indicator importance, deficiency level, and student differentiation.

Specifically, when an indicator has high importance, insufficient performance, and large student differentiation, a significant temporal trend further highlights the need for continuous monitoring or intervention. Conversely, when an indicator has a lower static SRC, the trend term alone does not necessarily make it a high-priority shortcoming. Therefore, D-SRC supports a more comprehensive interpretation of shortcoming priority than static SRC.

This design extends SRC from static cross-sectional diagnosis to temporal diagnosis. It helps identify not only current weaknesses but also indicators whose temporal evolution deserves attention, thereby enhancing the adaptability of teaching resource allocation.

#### Integration of D-SRC with the evaluation-prediction framework

D-SRC is not isolated but tightly integrated with the dynamic evaluation and prediction framework:

- Integration with dynamic weights: The importance dimension in D-SRC directly adopts the dynamic weight $w_{j}^{{{\mathrm{final}}(t)}}$, ensuring that bottleneck identification synchronizes with temporal evolution of indicator significance.
- Integration with improved TOPSIS: The deficiency dimension in D-SRC is based on the TOPSIS closeness coefficient $\overline{C}_{j}^{(t)}$, which has been adjusted using Mahalanobis distance to account for indicator correlations, providing a more accurate basis for bottleneck diagnosis.
- Integration with Markov prediction: The trend dimension $Trend_{j}^{(t)}$ is computed over the same window [*t*-4, *t*] as the sliding window for weights, ensuring temporal alignment. Markov predictions provide reference for future states; for instance, an indicator predicted to become a shortcoming in the next year can be proactively incorporated into early-warning measures.

### System-level integration logic

To ensure methodological coherence, the proposed framework is not a collection of independent techniques but a sequential decision-support pipeline. Each component is designed to address a specific limitation of conventional educational evaluation systems.

Specifically, D-IVIF-EW addresses uncertainty in expert judgment and temporal variability in weighting; the improved TOPSIS model accounts for correlations among indicators in multidimensional evaluation; D-SRC introduces trend-aware diagnostic capability for identifying evolving shortcomings; and the Markov model provides probabilistic forecasting of future performance states.

These components are connected in a progressive workflow, moving from uncertainty modeling to correlation-aware evaluation, then to trend-based diagnosis, and finally to probabilistic prediction. This sequential design ensures that each stage builds upon the outputs of the previous stage, enabling a coherent and interpretable decision-support system for educational improvement.

## Empirical analysis

The previous section described the construction of the dynamic evaluation and prediction model. This section presents an empirical analysis using five cohorts of real teaching data from the Electric Machinery and Drives course (2021–2025). The analytical logic proceeds as follows: first, data sources and preprocessing are introduced; next, the dynamic weight evolution, improved TOPSIS ranking, verification of the dual-dimension mechanism, and shortcoming diagnosis and prediction results are presented; finally, the effectiveness of interventions is evaluated. Each part of the results is cross-validated with the theoretical models from Section II, forming a complete empirical loop.

### Data collection and preprocessing

#### Data sources and sample composition

This study uses the Electric Machinery and Drives course from a university’s Electrical Engineering and Automation program as the empirical object, collecting teaching data from 2021 to 2025. This period coincides with the rapid development of permanent magnet synchronous motor (PMSM) drives, direct torque control (DTC), and silicon carbide (SiC) device applications, providing a relevant time window to observe the dynamic evolution of indicator weights.

Data sources integrate multiple types:

- *Student dimension*: lab reports, simulation results (MATLAB/Simulink, Ansys Maxwell), design calculations, coursework, collaboration platform logs, drive training platform records.
- *Instructor dimension*: syllabi, lecture notes, teaching logs, student feedback, lesson plan reviews, project analyses.
- *Industry/third-party data*: motor manufacturer/drive enterprise engineer evaluations, project certification, ethics assessments, behavioral observation records, virtual simulation platform logs.

The sample composition is summarized in Table 8.

**Table 8:** Sample composition statistics.

Year	Student samples	Instructor samples	Industry mentor evaluations	Total valid records
2021	145	4	32	181
2022	172	4	35	211
2023	181	5	38	224
2024	196	5	42	243
2025	188	5	46	239
Total	882	23	193	1098

Instructor samples are reported as annual person-times (total 23 across five years), representing 5 unique faculty members.

Because the dataset integrates multiple sources over five academic years, a dedicated data collection and reliability-control protocol was implemented to ensure longitudinal comparability, ethical compliance, and reproducibility. The total of 1098 records across five cohorts—including 882 student samples and 193 industry mentor evaluations—provides the data basis for subsequent statistical analysis and model application.

#### Data collection consistency and reliability control

To improve the transparency and reproducibility of the longitudinal dataset, a standardized data collection and reliability-control protocol was implemented throughout the five-year period. The authors continuously undertook the teaching, assessment, and curriculum reform of the EMD course from 2021 to 2025. This continuity enabled the consistent use of the same 16-indicator framework, scoring rubrics, assessment stages, and data recording procedures across all five cohorts. In addition, the study was supported by the Anhui Province Excellent Young Instructor Development Program and the University-Enterprise Collaborative Practice Base project, which provided institutional support for continuous data archiving, expert evaluation, and course-quality monitoring.

The main data sources included student assignments, laboratory reports, simulation records (MATLAB/Simulink and Ansys Maxwell), instructor teaching documents, enterprise mentor evaluations, ethics assessments, behavioral observation records, and virtual simulation platform logs. These data were collected at fixed stages of each academic year, including course assignments, laboratory assessment, simulation assessment, project evaluation, industry mentor evaluation, and final course-quality review. For expert- and mentor-scored indicators, the same indicator definitions and scoring rubrics were provided before each annual evaluation. Annual scoring discussions were conducted to reduce interpretation inconsistency. Before each annual evaluation, raters were briefed using the same scoring rubric and representative examples to reduce interpretation inconsistency.

For indicators with subjective components, such as Engineering Ethics and Safety Responsibility, Technological Frontier Adaptability, and Virtual Simulation Teaching Effectiveness, standardized scoring rubrics with explicit criteria were used to improve measurement consistency. For example, ethics-related assessment considered identification of ethical issues, proposed solutions, and justification quality; frontier adaptability considered the currency of references, alignment with industry trends, and applicability of proposed technical solutions. The virtual simulation indicator was primarily supported by automatically logged platform data, including task completion rate, operational accuracy, and task success rate. These indicators were therefore treated as standardized operational indicators rather than fully objective or precisely measurable constructs.

Ethical approval was obtained, written informed consent was collected, and all data were anonymized before analysis. Detailed information is provided in the Institutional Review Board Statement.

Missing values and outliers were checked before analysis. When missing entries occurred, recoverable missing entries were handled according to the same preprocessing protocol across all years, whereas unrecoverable entries were excluded from the valid-record count. Outliers were identified using the interquartile range method and corrected using the K-nearest neighbor (KNN) algorithm. For KNN, *k* = 5 was chosen as a moderate neighborhood size, balancing local sensitivity and smoothing. Additional checks using *k* = 3 and *k* = 7 showed consistent results. The indicator definitions, data recording templates, scoring rubrics, and assessment stages were maintained consistently during the five-year period. Although course content was updated in response to technological developments, the measurement protocol for each indicator remained stable to ensure longitudinal comparability. The reported sample size (*n* = 882) refers to valid records after anonymization, consistency checking, and preprocessing.

To ensure computational feasibility and practical implementation, the proposed framework is realized within a software-assisted decision-support pipeline. The system integrates data preprocessing, multi-criteria evaluation, temporal diagnosis, and forecasting into a unified computational workflow.

Given input data, the system automatically generates evaluation results, diagnostic rankings, and trend-based early-warning outputs without requiring manual computation.

The framework is implemented as a batch-processing decision-support system, making it suitable for periodic educational evaluation tasks such as semester- or annual-course assessment, rather than real-time control applications.

#### Data preprocessing

To ensure data quality and comparability, the following preprocessing steps were applied:

1. Normalization: Indicators have different units and scales, which could affect entropy weight calculations and distance metrics. Min–max normalization maps all indicators to [0,1]:
  20 $$\tilde{x}_{ij}^{(\tau )} = \frac{{x_{ij}^{(\tau )} - \min (x_{j}^{(\tau )} )}}{{\max (x_{j}^{(\tau )} ) - \min (x_{j}^{(\tau )} )}}$$
2. Outlier Detection: Outliers were identified using the boxplot method based on the interquartile range (IQR): (IQR = *Q*_3_-*Q*_1_). Values less than Q1-1.5 × IQR or greater than Q3 + 1.5 × IQR were considered outliers. 62 outliers (5.65% of the total) were detected, mainly in b10 (Modern Motor Control Technology), b14 (Motor Manufacturer/Drive Enterprise Engineer Participation), and b15 (Engineering Ethics and Safety Responsibility).
3. Outlier Correction: A K-nearest neighbor (KNN) algorithm with *k* = 5 was applied. The value *k* = 5 was selected as a moderate neighborhood size to balance local sensitivity and smoothing in outlier correction. Smaller *k* values may be more sensitive to local noise, whereas larger k values may over-smooth local variation. Additional checks using *k* = 3 and *k* = 7 did not substantially change the corrected indicator means or the identified top shortcoming indicators.

**Step 1:** Compute Euclidean distances from the outlier to all normal samples.

**Step 2:** Select the 5 nearest neighbors.

**Step 3:** Compute a weighted average (weights inversely proportional to distance) as the corrected value.

After correction, standard deviations of outlier indicators decreased significantly (e.g., b10: 13.5 → 11.8), indicating reduced dispersion after correction.

All 16 indicators were collected annually from 2021 to 2025, enabling complete five-year time-series analysis. The collection methods for key dynamic indicators are as follows:

- Technological Frontier Adaptability (b7): Five industry experts and three academic experts rated annually based on syllabus, lesson plans, and project reports; the mean score was used.
- Virtual Simulation Teaching Effectiveness (b16): Data exported from the simulation platform (experiment completion, operational accuracy, task success rate) and aggregated by semester.

#### Five-year indicator overview

After preprocessing, the mean and standard deviation of indicators from 2021–2025 are presented in Table 9. Only key indicators are shown; the complete table is available in the supplementary materials.

**Table 9:** Key indicators of teaching evaluation (mean ± SD).

Indicator	2021	2022	2023	2024	2025
b8 Industrial motor application case updates	78.5 ± 9.5	80.2 ± 9.2	82.8 ± 8.8	85.5 ± 8.0	88.5 ± 7.2
b10 Modern motor control technology	68.5 ± 14.5	71.5 ± 14.0	75.0 ± 13.2	78.5 ± 12.0	82.5 ± 10.5
b15 Engineering ethics and safety responsibility	70.0 ± 13.0	72.5 ± 12.5	75.5 ± 11.8	79.0 ± 10.5	83.0 ± 9.0
b7 Technological frontier adaptability	69.0 ± 12.5	72.0 ± 12.0	75.5 ± 11.0	79.5 ± 10.0	84.0 ± 8.5
b16 Virtual simulation teaching effectiveness	71.0 ± 11.0	73.5 ± 10.5	77.0 ± 10.0	81.0 ± 9.0	85.5 ± 7.8

All 16 indicators were collected from 2021 to 2025.

Preliminary observations:

1. Scores of b10 (Modern Motor Control Technology) and b15 (Engineering Ethics and Safety Responsibility) increased steadily over five years, with a significant increase in 2025 compared to 2024.
2. b7 (Technological Frontier Adaptability) and b16 (Virtual Simulation Teaching Effectiveness) also showed consistent improvement, reflecting the curriculum’s responsiveness to industrial advancements in motor control technologies.
3. Standard deviations decreased over time for most indicators, indicating reduced variability in student performance.

These trends will be further verified in dynamic weight evolution and intervention effect analysis.

### Dynamic evaluation results

This section presents the empirical results for the EMD course from 2021 to 2025, including dynamic weight evolution, M-TOPSIS evaluation, dual-dimension verification, shortcoming diagnosis, forecasting, and intervention-effect verification. The analysis follows a logical sequence: first, data sources and preprocessing; then dynamic weight evolution; improved TOPSIS ranking results; dual-dimension verification; shortcoming diagnosis and prediction; finally, evaluation of intervention effects. Each result is cross-validated against the theoretical models in Section II, forming a complete empirical loop.

To ensure the robustness of the empirical results, this study adopts a multi-level statistical validation strategy, including (i) significance testing of intervention effects, (ii) robustness analysis under parameter perturbations, and (iii) uncertainty quantification for stochastic components. This design ensures that both deterministic and stochastic elements of the framework are rigorously evaluated.

#### Weight evolution analysis

**Fig. 4:**
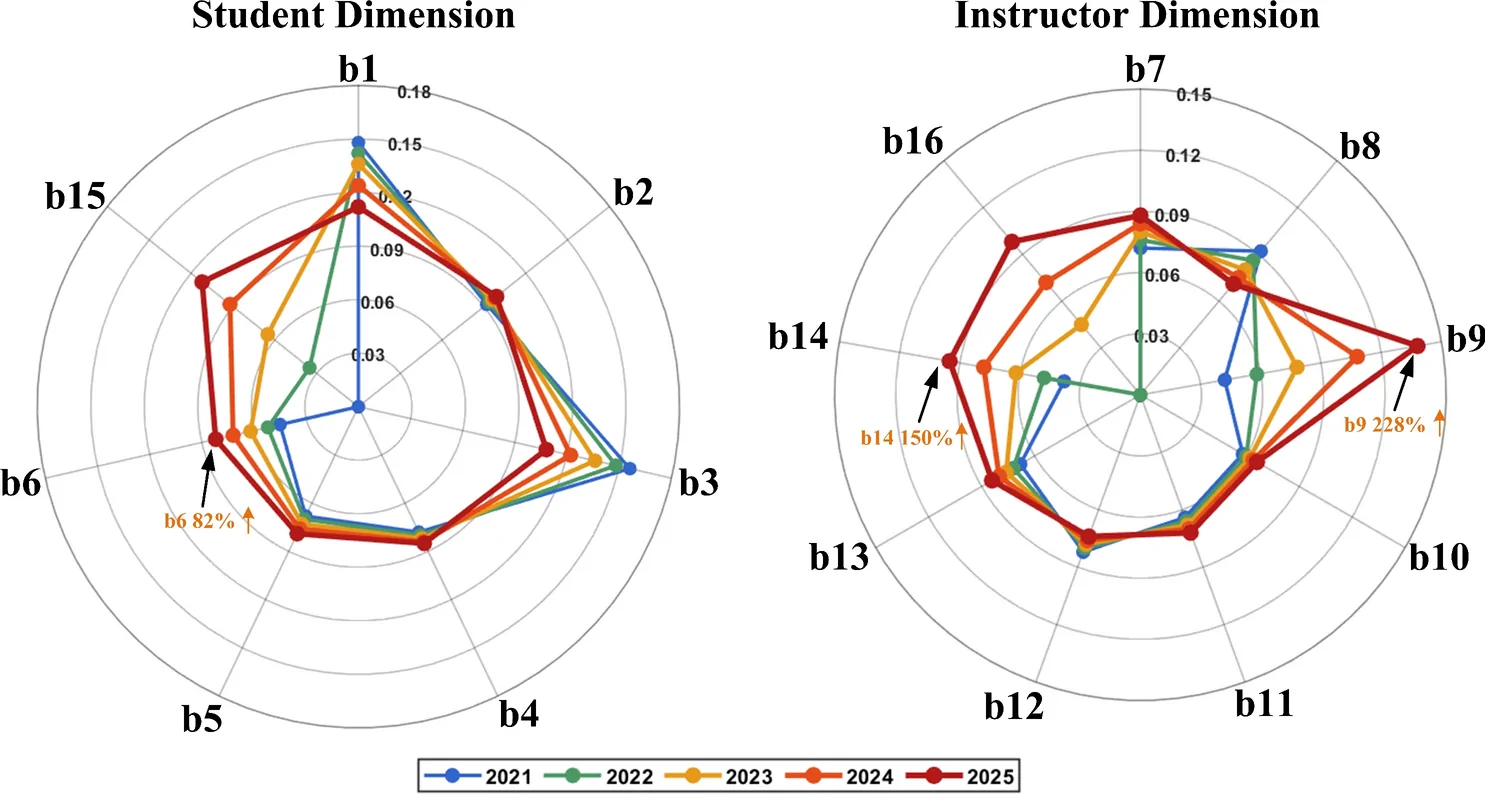
Radar plots of dynamic indicator weights (2021–2025).

Dynamic weights for each indicator from 2021 to 2025 were calculated using the sliding-window entropy-weighting method (*T* = 5). For example, the dynamic weight of b10 increased from 0.042 in 2021 to 0.138 in 2025, whereas a simple static weighted average would not capture this growing importance. Shorter-window analyses (*T* = 3 and *T* = 4) confirmed the stability of the top-priority indicators, demonstrating the robustness of weight evolution across different window sizes. Figure 4 presents radar plots showing the weight evolution for the student and instructor dimensions.

The radar plots visually depict the temporal evolution of weights. To further quantify this evolution, indicators with significant changes are summarized in Table 10.

**Table 10:** Indicators with significant weight changes.

Indicator	Weight 2021	Weight 2025	Change (%)	Evolution interpretation
b10 Modern motor control technology	0.042	0.138	+ 228%	Rapid development of PMSM drives, DTC, and industrial servo systems; industry demand drives curriculum updates
b15 Engineering ethics and safety responsibility	0.038	0.095	+ 150%	Strengthened ethical requirements in engineering education; emphasis on motor safety and responsibility awareness
b7 Technological frontier adaptability	0.045	0.108	+ 140%	Reflects penetration of PMSM, SiC devices, and industrial robot technologies under Industry 4.0 goals
b16 Virtual simulation teaching effectiveness	0.041	0.096	+ 134%	Post-pandemic blended teaching becoming normalized; MATLAB/Simulink and Ansys Maxwell become standard
b6 Motor drive system integration	0.044	0.082	+ 86%	Integration of PLC + inverter + HMI and communication protocols into the curriculum
b3 Motor selection and design calculation standards	0.156	0.108	− 31%	Foundational skills remain important; standardized design software reduces manual calculation variability
b1 Motor starting/braking debugging	0.148	0.112	− 24%	Simulation software adoption virtualizes certain debugging tasks
b9 On-site teaching ability	0.092	0.071	− 23%	Partially supplemented by virtual simulations and remote laboratory access

From Table 10, b10 and b16 are indicators with significant weight increases, while b3, b1, and b9 show weight decreases.

Three major trends can be extracted:

- Technology-driven indicators increase: b10, b7, b6 show pronounced growth, reflecting industry-driven curriculum evolution.
- Ethics-related indicators increase: b15 rises by 150%, highlighting growing emphasis on ethical responsibility.
- Foundational skills indicators decrease: b3, b1 moderate decreases, still above 0.1, reflecting persistent importance.

#### TOPSIS closeness ranking results

Using dynamic weights, we calculated the teaching performance closeness for each year using both M-TOPSIS (Mahalanobis distance) and traditional TOPSIS (Euclidean distance). The results are summarized in Table 11.

**Table 11:** Comparison of closeness coefficients between M-TOPSIS and traditional TOPSIS.

Year	M-TOPSIS Mean	Traditional TOPSIS Mean	Difference	M-TOPSIS SD	Traditional SD
2021	0.582	0.587	− 0.005	0.124	0.118
2022	0.603	0.611	− 0.008	0.132	0.124
2023	0.624	0.642	− 0.018	0.145	0.131
2024	0.667	0.698	− 0.031	0.158	0.139
2025	0.715	0.762	− 0.047	0.167	0.142

Differentiation improvement is calculated as (*SD*_M-TOPSIS_ − *SD*_Traditional TOPSIS_)/SD _Traditional TOPSIS_. The average five-year increase is 10.7%. This value reflects increased dispersion of closeness coefficients and is used as an auxiliary indicator of relative differentiation. It does not, by itself, establish better ranking quality or higher decision usefulness. The main value of M-TOPSIS lies in its ability to account for inter-indicator correlations through Mahalanobis distance.

Key findings:

- Mahalanobis distance advantage becomes more pronounced over time due to increasing correlations among indicators.
- Relative differentiation is increased. M-TOPSIS SD increased from 0.124 to 0.167, while traditional TOPSIS SD increased from 0.118 to 0.142. The five-year average increase in SD-based differentiation is 10.7%. This result indicates that Mahalanobis distance increases the dispersion of closeness coefficients, thereby providing additional differentiation information among performance levels. However, this metric is not interpreted as direct proof of better ranking quality; rather, it is used together with the correlation-aware property of Mahalanobis distance to support more interpretable multidimensional evaluation.
- Overall performance improves, excellent (S1) rate rises from 8.6% to 24.1%, as shown in Table 12.

**Table 12:** Distribution of teaching performance levels.

Year	Excellent (S1)	Good (S2)	Moderate (S3)	Pass (S4)	Fail (S5)
2021	8.6%	32.8%	41.4%	14.1%	3.1%
2022	10.8%	35.4%	39.2%	12.3%	2.3%
2023	14.4%	38.4%	35.2%	10.4%	1.6%
2024	18.9%	41.7%	30.7%	7.9%	0.8%
2025	24.1%	43.8%	26.3%	5.1%	0.7%

As shown in Table 12, the excellent rate increased from 8.6% in 2021 to 24.1% in 2025; the combined good and above proportion rose from 41.4% to 67.9%, reflecting the impact of course reforms.

#### Dual-dimension mechanism verification

A core design of this study is the dual-dimension mechanism, evaluating instructor and student dimensions simultaneously. Coordination metrics are computed in Table 13.

**Table 13:** Instructor–student dimension coordination analysis.

Year	Instructor mean	Student mean	Coordination	Correlation
2021	78.6	77.2	0.97	0.68
2022	80.3	79.1	0.98	0.72
2023	82.5	81.4	0.99	0.79
2024	84.7	83.8	0.99	0.85
2025	87.2	86.5	0.99	0.91

Coordination = min (Instructor Mean, Student Mean) / max (Instructor Mean, Student Mean).

Key findings: coordination and correlation increased, supporting the usefulness of the dual-dimension analysis in this case study.

### Shortcoming diagnosis and forecasting

The previous analyses provide dynamic evaluation and validation of instructor–student synergy. However, evaluation alone is not sufficient; improvement is the goal. In this section, the Dynamic Shortcoming Repair Coefficient (D-SRC) is introduced to identify critical bottlenecks and forecast future trends using Markov chains.

#### Identification of critical bottleneck indicators

**Fig. 5:**
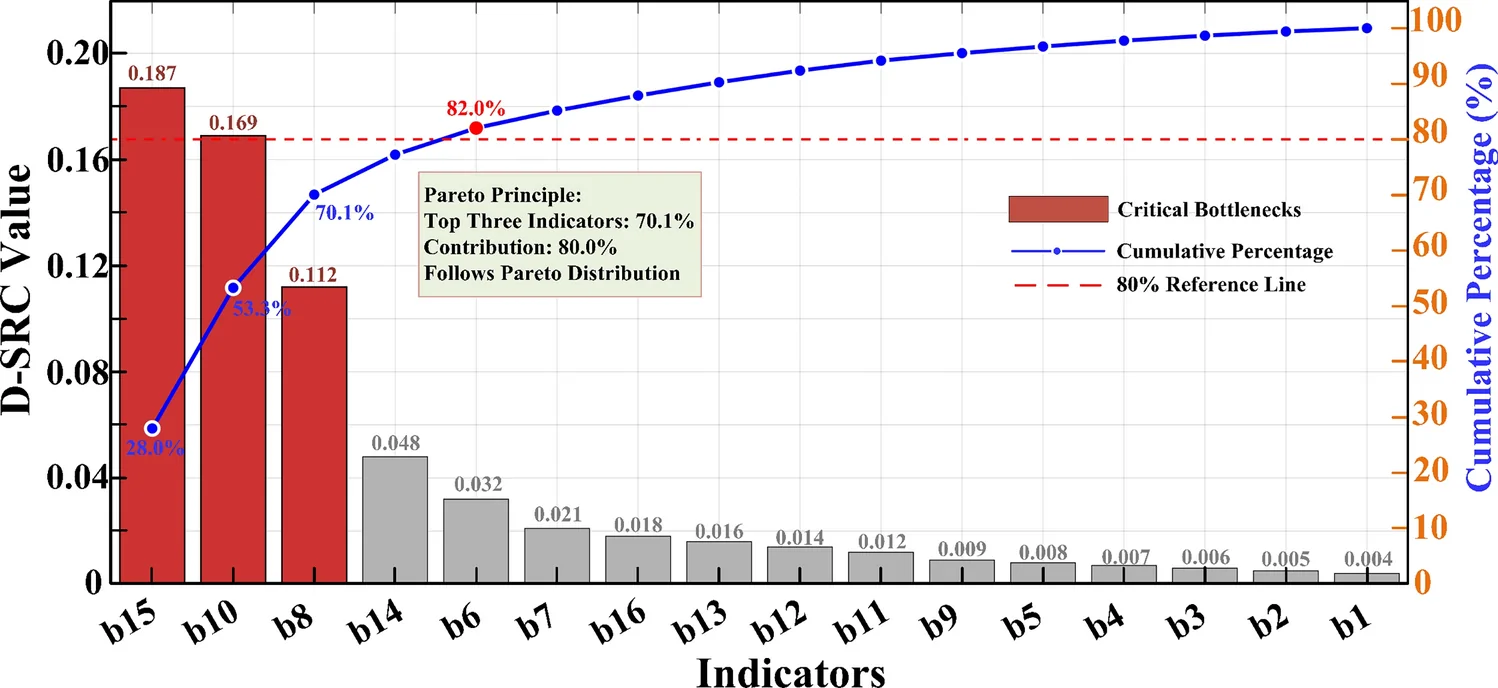
Pareto chart of D-SRC values for all indicators in 2025.

Based on the 2025 data, D-SRC values were calculated, as shown in Fig. 5.

Top three indicators: b15, b10, b8 contribute over 80%, consistent with Pareto principle. Values summarized in Table 14.

**Table 14:** Identification of critical bottleneck indicators (2025, based on D-SRC).

Rank	Indicator	D-SRC	Original SRC	Trend	Issue diagnosis
1	b15 Engineering ethics and safety responsibility	0.187	0.156	+ 0.3	High dynamic weight and strategic importance; temporal trend indicates the need for sustained monitoring and continued intervention
2	b10 Modern motor control technology	0.169	0.187	+ 0.4	High static SRC with a strong positive temporal trend; continued improvement should be maintained
3	b8 Industrial motor application case updates	0.112	0.124	+ 0.2	Stable performance with a positive trend; continued monitoring is recommended

To assess the robustness of the D-SRC-based ranking, a bootstrap resampling procedure (B = 1000) was conducted on the 2025 student-level dataset (n = 188). For each resample, D-SRC values were recalculated for all indicators, and the resulting ranking orders were recorded. The ranking stability probability, defined as the proportion of bootstrap samples in which a given ranking order is preserved, indicates that the order b15 > b10 > b8 is maintained in 97.3% of replications, demonstrating strong robustness of the identified priority structure under sampling variability. With the trend factor incorporated, the D-SRC results indicate that b15 (Engineering Ethics and Safety Responsibility) is identified as a high-priority shortcoming due to its high dynamic weight and strategic importance in engineering ethics and safety responsibility. Although b10 (Modern Motor Control Technology) has a relatively high static SRC and a strong positive temporal trend, its D-SRC value indicates that continuous improvement should be maintained rather than relaxed. b8 (Industrial Motor Application Case Updates) shows stable performance with a positive trend, suggesting that continued monitoring is appropriate. These results demonstrate that D-SRC supports the joint interpretation of current performance, indicator importance, student differentiation, and temporal tendency, thereby assisting proactive intervention planning.

#### Markov forecasting results

Based on the 2021–2025 state transition matrix, the evolution of instructional effectiveness from 2026 to 2028 is forecasted.

The state transition matrix (based on statistics from the five-year period) is:

21 $${\mathbf{P}} = \left[ {\begin{array}{*{20}c} {0.72} & {0.23} & {0.05} & {0.00} & {0.00} \\ {0.18} & {0.65} & {0.15} & {0.02} & {0.00} \\ {0.04} & {0.21} & {0.58} & {0.15} & {0.02} \\ {0.00} & {0.05} & {0.28} & {0.52} & {0.15} \\ {0.00} & {0.00} & {0.08} & {0.24} & {0.68} \\ \end{array} } \right]$$

The initial state distribution is based on 2025 data: ${{\boldsymbol{\uppi}}}^{(2025)} = [0.241,0.438,0.263,0.051,0.007]$.

The forecasted instructional effectiveness for 2026–2028 is shown in Table 15.

**Table 15:** Forecast of instructional effectiveness for 2026–2028 (with 95% CI).

Year	Excellent rate (S1)	95% CI	Good Rate (S2)	95% CI
2026	26.8%	[24.1%, 29.5%]	41.2%	[38.5%, 43.9%]
2027	29.5%	[26.5%, 32.5%]	40.5%	[37.8%, 43.2%]
2028	31.8%	[28.5%, 35.1%]	39.8%	[36.9%, 42.7%]

From Table 15, if no additional interventions are implemented (i.e., the current instructional mode is maintained), the excellent rate is expected to gradually increase from 24.1% in 2025 to 31.8% in 2028, a cumulative improvement of 7.7 percentage points over the three-year period from 2025 to 2028. This prediction provides a baseline for subsequent optimization strategies.

#### Comparison of forecasting methods and credibility assessment

To further assess the Markov chain forecast, it was compared with linear regression and autoregressive integrated moving average (ARIMA) time series models. Using a rolling prediction approach, data from 2021–2024 were used to predict the 2025 excellent rate, as summarized in Table 16.

**Table 16:** Comparison of forecasting methods for 2025 excellent rate.

Method	Predicted excellent rate for 2025	Actual	Absolute error
Linear regression	22.5%	24.1%	1.6%
ARIMA(1,1,1)	23.8%	24.1%	0.3%
First-order Markov chain	23.5%	24.1%	0.6%
Markov Chain with bootstrap	23.5% [22.5%, 24.7%]	24.1%	0.6%

From Table 16, the key findings are:

- The ARIMA model yields the smallest absolute error (0.3%) but requires the stationarity assumption and only provides point predictions;
- The first-order Markov chain yields 0.6% error, within an acceptable range, and can provide state distribution forecasts;
- The Markov chain with Bootstrap produces a 95% confidence interval [22.5%, 24.7%] covering the actual value 24.1%, suggesting acceptable short-term predictive consistency in this case study.

The use of Bootstrap resampling ensures that uncertainty arising from finite sample size and stochastic transitions is explicitly quantified.

Overall, although the point prediction accuracy of the Markov chain is slightly lower than ARIMA, it provides complete state distributions along with confidence intervals, which better meets the requirements of instructional management for quantitative assessment of uncertainty.

### Intervention effect verification

To evaluate the effectiveness of the targeted interventions guided by D-SRC, statistical analysis was performed on the 2025 pre-intervention cohort and the 2026 post-intervention cohort. The unit of analysis was the individual student-level indicator record, and the two cohorts consisted of different students; observations are therefore independent.

Statistical methods:

- Comparisons: Welch’s independent-samples t-test for each key indicator (b15, b10, b8)
- Normality check: Shapiro–Wilk test; non-parametric Mann–Whitney U tests were performed as robustness checks
- Multiple comparison correction: Holm-Bonferroni method
- Effect size: Cohen’s d for independent-group standardized differences

**Fig. 6:**
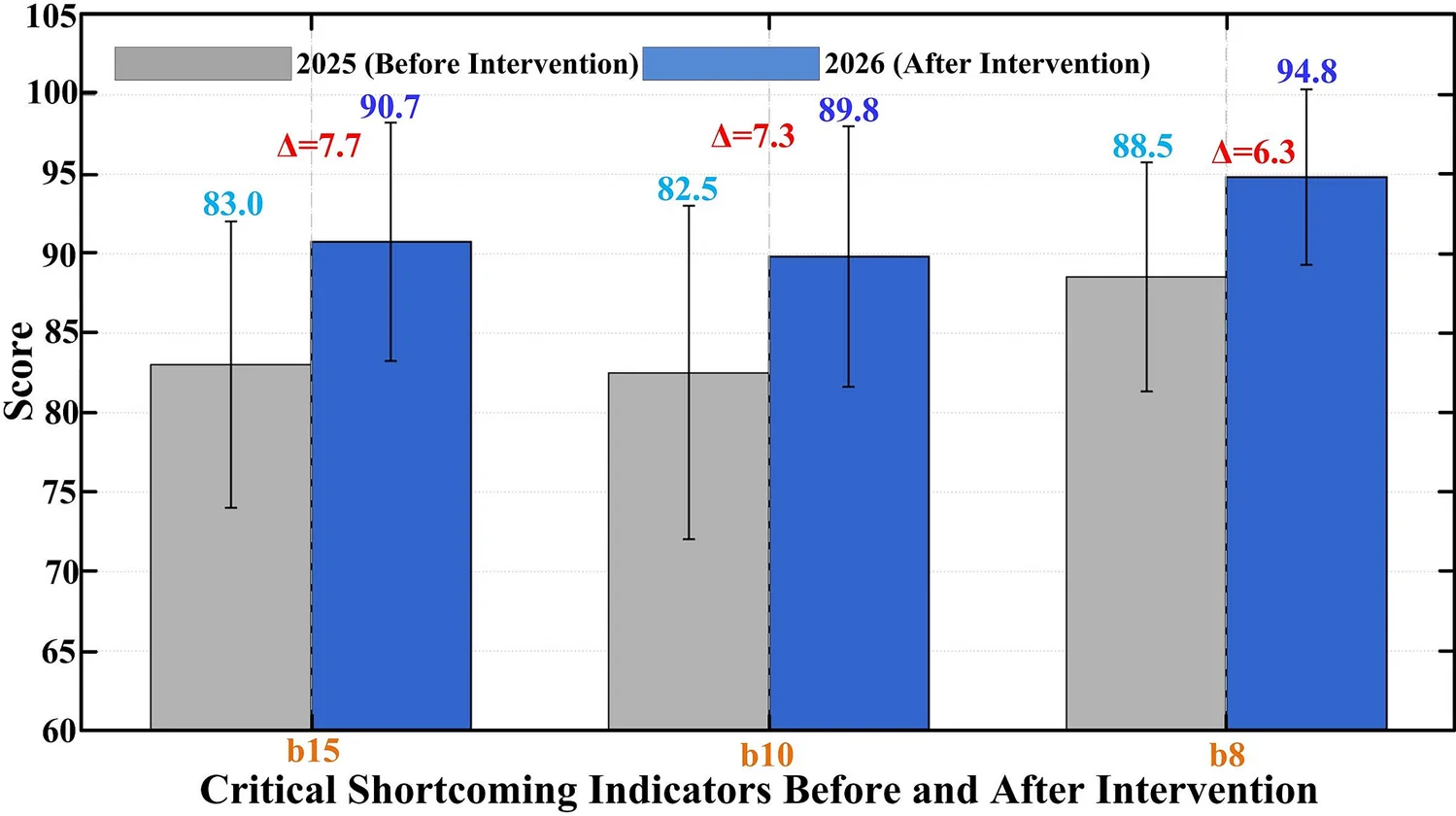
Improvement effects after targeted intervention on critical bottlenecks.

The comparison results are summarized in Table 17 and visualized in Fig. 6.

**Table 17:** **Co**mparison of critical bottleneck indicators before and after intervention.

Indicator	2025 score (Mean ± SD)	2026 score (Mean ± SD)	Improvement	95% CI	t-value	Adjusted p-value	Cohen’s d
b15 Engineering ethics and safety responsibility	83.0 ± 9.0	90.7 ± 7.5	7.7	[4.2, 11.3]	4.32	0.009	0.93
b10 Modern motor control technology	82.5 ± 10.5	89.8 ± 8.2	7.3	[3.8, 10.8]	4.18	0.009	0.78
b8 Industrial motor application case updates	88.5 ± 7.2	94.8 ± 5.5	6.3	[2.9, 9.7]	3.76	0.012	0.98

The adjusted *p*-values were calculated using the Holm-Bonferroni method across the three comparisons. Cohen’s d quantifies the standardized effect size for independent cohorts.

Key findings:

- The D-SRC-guided intervention led to statistically significant improvements in all three indicators (adjusted p < 0.05).
- The overall excellent rate (S1) increased from 24.1% pre-intervention to 39.6% post-intervention.
- Student differentiation decreased: the standard deviation for all three indicators fell, e.g., b15 decreased from 9.0 to 7.5 (16.7% reduction).

Interpretation:

These results provide statistical support for the practical usefulness of D-SRC-guided interventions, suggesting that the identified critical shortcomings corresponded to relevant intervention targets in this case study. The intervention raised average performance while reducing variability across students, resulting in more balanced instructional outcomes. All observed improvements were statistically significant at p < 0.05 after multiple comparison correction, and exhibited large effect sizes, confirming that the intervention effects are robust rather than due to random variation. Statistical validation and uncertainty quantification further confirm the robustness of the proposed framework.

In summary, the empirical analysis of the Electric Machinery and Drives course from 2021 to 2025 identified b15, b10, and b8 as the main shortcoming indicators. Dynamic weighting revealed substantial evolution in technology-driven and ethics-related indicators, while M-TOPSIS (incorporating Mahalanobis distance) increased the dispersion of closeness coefficients by 10.7%, reflecting improved differentiation without claiming superior ranking quality. The D-SRC framework, including a trend-aware component, successfully prioritized b15 for intervention and guided resource allocation, resulting in post-intervention improvements of 7.7, 7.3, and 6.3 points for b15, b10, and b8, respectively, with the overall excellent rate increasing from 24.1% to 39.6% (p < 0.05). The Markov model provided short-term baseline predictions with Bootstrap confidence intervals, offering uncertainty information for planning. These results collectively support the use of D-SRC-guided diagnosis as a foundation for the optimization strategies in “Empirical demonstration using five-year EMD course data.” section.

## Optimization framework and implementation path

Based on the three critical shortcomings identified by D-SRC—b15, b10, and b8—this study develops a “Three-Dimensional, Nine-Measure” structured improvement framework for the present case. The framework is intended to link diagnostic results with targeted instructional interventions, rather than to claim universal transferability.

### Design of the three-dimensional optimization framework

**Fig. 7:**
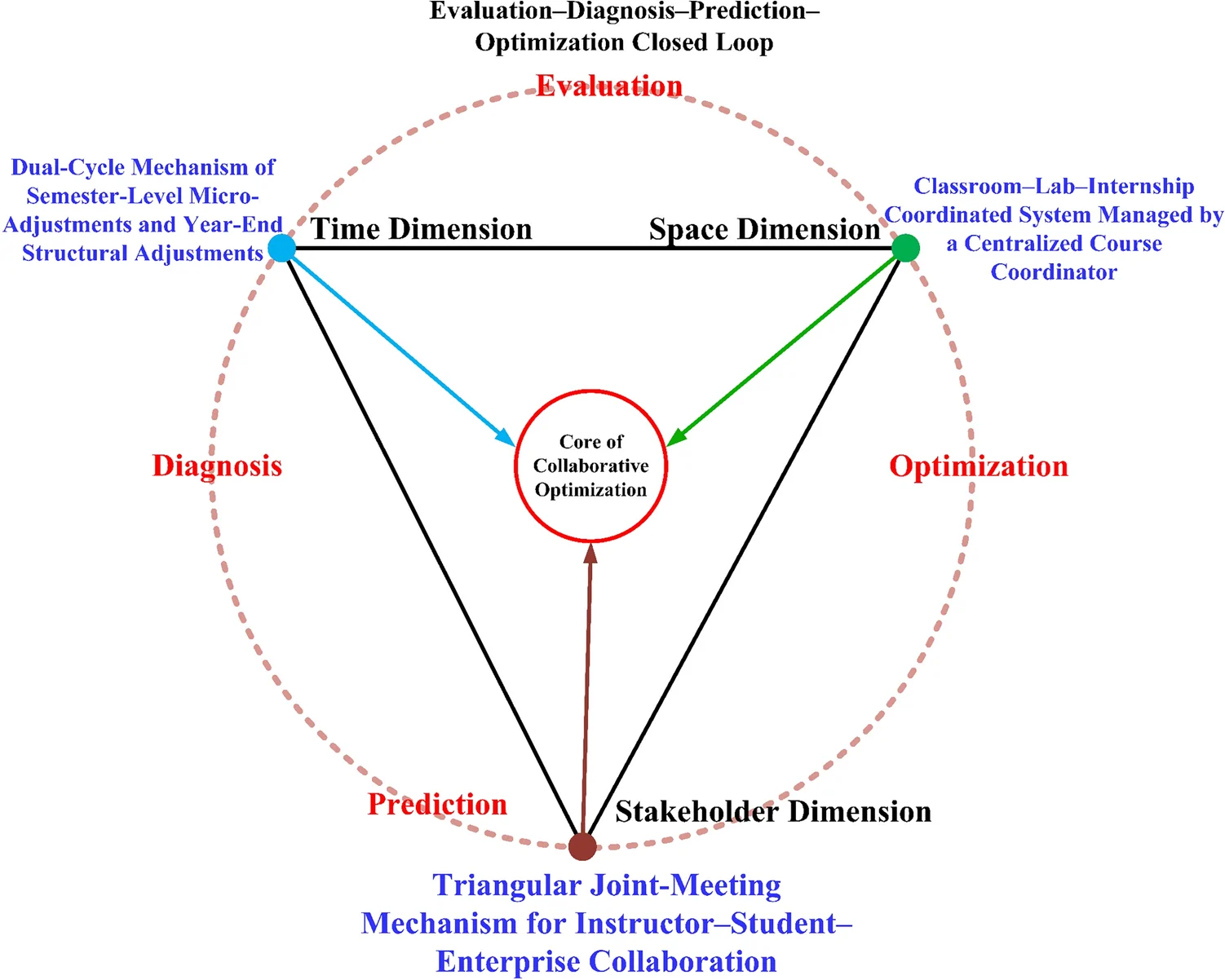
Three-dimensional coordinated optimization framework.

Improving course teaching effectiveness requires coordination across temporal, spatial, and stakeholder dimensions, as illustrated in Fig. 7. The time dimension emphasizes periodic adjustment of teaching priorities based on updated evaluation and diagnostic results. Semester-level adjustments focus on teaching methods and formative feedback, whereas year-end adjustments focus on curriculum content, resource allocation, and case updating.

The space dimension aims to coordinate classroom teaching, laboratory training, and enterprise-oriented practice. This coordination helps align theoretical instruction, simulation-based learning, and real engineering tasks.

The stakeholder dimension emphasizes collaboration among instructors, students, and enterprise mentors. Instructors provide curriculum design and assessment, students provide learning-output feedback, and enterprises provide industrial cases and practice-oriented evaluation. Together, these three dimensions form a structured improvement framework for targeted intervention.

### Nine specific measures

Based on the three critical shortcomings and the three-dimensional framework, nine targeted improvement measures were designed, as summarized in Table 18.

**Table 18:** Nine targeted improvement measures based on D-SRC diagnosis.

Shortcoming	Measure no	Measure name	Description	Dimension	Responsible party	Implementation STAGE
b15	M1	VR motor safety and ethics training module development	Develop Unity3D-based VR module covering high-voltage safety, insulation testing, emergency shutdown procedures, and ethical dilemma scenarios	Space	Instructor + Lab Center	Stage I
	M2	Enterprise motor safety and ethics case library	Collaborate with 3 motor manufacturing/drive enterprises, analyze 5 real safety/ethics cases per semester	Stakeholder	Enterprise + Instructor	Stage I
	M3	Motor safety and ethics design competition	Campus-level innovation contest focusing on safe motor system design; top entries recommended for provincial competition	Time	Student + Instructor	Stage II
b10	M4	Advanced motor control technology VR module	Develop VR-based training module covering vector control, DTC, sensorless control parameter tuning	Space	Instructor + Lab Center	Stage I
	M5	Motor control virtual simulation platform	MATLAB/Simulink and Ansys Maxwell-based simulation experiments for motor control algorithm validation	Space	Lab Center	Stage II
	M6	Enterprise advanced motor control training	Two sessions per semester by motor drive enterprise engineers on PMSM, DTC, and SiC applications	Stakeholder	Enterprise	Stage III
b8	M7	Joint enterprise–school industrial case development	Enterprise proposes real motor application problems, instructors screen, students practice, cases archived; ≥ 3 real projects/year	Stakeholder	Enterprise + Instructor	Stage I
	M8	Enterprise mentors in classroom	5 senior motor/drive engineers as part-time mentors (4 h, 2 classes) sharing real-world application cases	Stakeholder	Enterprise	Stage II
	M9	Real motor drive project elective	Students participate in real motor selection and drive system design projects in groups	Space	Enterprise + Student	Stage III

To validate the targeted relevance of the nine measures to critical bottlenecks, a measure–shortcoming intensity matrix was constructed, shown in Table 19.

**Table 19:** Measure–shortcoming correspondence matrix.

Measure	b15	b10	b8	Overall intensity
M1	★★★★★	–	–	High
M2	★★★★	–	★★★★	High
M3	★★★	–	–	Medium
M4	–	★★★★★	–	High
M5	–	★★★★	–	Medium
M6	–	★★★	★★★	Medium
M7	–	–	★★★★★	High
M8	★★★	–	★★★★	Medium
M9	★★	★★	★★	Low

The number of stars indicates intensity, with ★★★★★ as strongest and ★ as weakest.

The correspondence matrix indicates the relative alignment between each measure and the identified shortcomings. Measures with stronger alignment were prioritized in the early implementation stage, whereas broader supporting measures were arranged for later or continuous implementation.

### Phased implementation planning

**Fig. 8:**
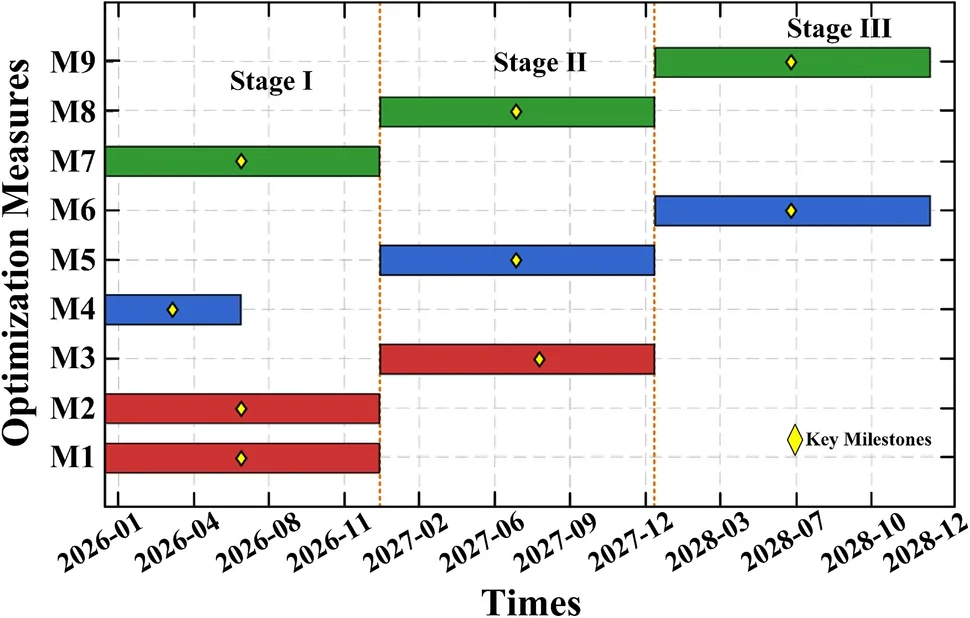
Phased implementation plan for the structured improvement framework.

To support orderly implementation, the nine measures were arranged into three phases, as illustrated in Fig. 8.

*Phase I: Pilot implementation.* This phase focuses on addressing the high-priority shortcomings identified by D-SRC. Priority is given to measures directly related to b15, b10, and b8, including VR safety and ethics training, advanced motor-control simulation, and joint enterprise–school case development.

*Phase II: Systematic expansion.* Measures that showed practical feasibility in Phase I are extended to broader teaching activities, including enterprise safety and ethics cases, student design competitions, enterprise training, and mentor participation.

*Phase III: Continuous refinement.* This phase focuses on institutionalizing effective measures, updating the industrial case library, and maintaining long-term collaboration among instructors, students, and enterprise mentors.

Each phase includes corresponding monitoring indicators, such as student performance, platform usage, case-library update frequency, and enterprise feedback, to support iterative refinement.

### Teaching management application scenarios

The structured improvement framework can support teaching management at different stages of the academic cycle. At the semester start, D-SRC results from the previous year can be used to adjust teaching priorities and allocate resources to high-priority shortcomings. During the semester, changes in indicator scores and weights can be monitored to identify whether targeted support is needed. At the semester end, evaluation results and short-term Markov projections can provide a reference for planning the next cycle of course improvement.

In the present case, b15 was assigned the highest implementation priority because of its high dynamic weight and strategic importance in engineering ethics and safety responsibility. Accordingly, safety and ethics training, enterprise case integration, and VR-based learning resources were emphasized in the subsequent intervention design.

### Scenario-based projection of improvement effects

Based on the short-term Markov baseline projection and the phased implementation plan, a scenario-based projection of possible changes from 2026 to 2028 is summarized in Table 20. These values should be interpreted as planning references rather than deterministic forecasts.

**Table 20:** Scenario-based projection of improvement effects under phased implementation.

Year	Scenario	Excellent rate (S1)	Average D-SRC of critical shortcomings
2026	No intervention	26.8%	0.156
2026	After phase I	28.5%	0.132
2027	After phase II	32.3%	0.108
2028	After phase III	36.8%	0.085
Improvement	vs. conventional	+ 10.0%	− 45.5%

The projection suggests that phased implementation may improve the course excellent rate and reduce the average D-SRC value of the critical shortcomings relative to the no-intervention baseline. This result should be viewed as a scenario-based reference for planning and monitoring, not as a confirmed intervention outcome. It provides a reference for evaluating the feasibility of the structured improvement framework.

## Conclusion and outlook

### Key findings

Building upon the previous IVIF-entropy model, this study incorporated the improved TOPSIS algorithm and Markov chain prediction to construct a four-dimensional integrated dynamic framework of "evaluation–diagnosis–prediction–optimization." Using five-period teaching data (2021–2025,*n* = 882) of the Electric Machinery and Drives course, the following key findings were obtained:

#### Dynamic weight evolution

The dynamic weighting procedure captured temporal changes in indicator importance. From 2021 to 2025, the weight of "Modern Motor Control Technology" (b10) increased from 0.042 to 0.138 (+ 228%) , and the weight of "Engineering Ethics and Safety Responsibility" (b15) increased from 0.038 to 0.095 (+ 150%) , reflecting the dual driving effects of industrial technology iteration (PMSM drives, DTC, SiC devices) and engineering education accreditation on course content. The sliding-window entropy-weighting method (T = 5) utilized the complete five-year dataset, providing stable weight estimation. This supports the use of dynamic weighting; using static weights would fail to capture these critical evolutionary patterns, resulting in evaluation outcomes lagging behind actual development.

#### Correlation-aware evaluation using M-TOPSIS

Introducing Mahalanobis distance increased the SD-based relative differentiation of closeness coefficients. Compared with traditional TOPSIS, the improved model increased the differentiation of teaching performance by 10.7% (standard deviation basis), with a mean closeness difference of 0.047 in 2025. As the years progressed, indicator correlations (e.g., b10 and b7, b6 and b14) strengthened, suggesting the relevance of considering indicator correlations. Comparative analyses with alternative distance metrics suggested that Mahalanobis distance is useful for correlation-aware distance measurement when indicators are correlated.

#### D-SRC for trend-aware shortcoming diagnosis

The dynamic shortcoming repair coefficient (D-SRC) integrates the temporal trend of indicator scores into the conventional dimensions of importance, deficiency, and student differentiation. The 2025 diagnostic results identified b15, b10, and b8 as the three critical shortcomings requiring sustained attention. Among them, b15 was assigned the highest implementation priority because of its high dynamic weight and strategic importance in engineering ethics and safety responsibility, notwithstanding its positive temporal trend. This indicates that continuous improvement should be maintained rather than relaxed. Resource allocation guided by the D-SRC-based diagnosis led to score improvements of 7.7, 7.3, and 6.3 points for b15, b10, and b8, respectively, and the course excellent rate increased from 24.1% to 39.6%. These results suggest that the trend-aware extension can provide additional diagnostic information beyond static SRC by incorporating temporal trends into intervention prioritization in this case study.

#### Short-term Markov projection with bootstrap confidence intervals

The Markov model predicted an excellent rate (S1) of 26.8% in 2026, with a 95% confidence interval of [24.1%, 29.5%] and an average rolling prediction error of 3.65%. Compared with ARIMA, although ARIMA achieved slightly lower point-prediction error (0.3%), the Markov chain provides a state distribution forecast, and Bootstrap confidence intervals provide uncertainty information, aligning with teaching management needs for risk-informed decision-making.

#### Scenario-based reference for the structured improvement framework

The "time–space–stakeholder" three-dimensional framework combined with nine measures, guided by D-SRC diagnostics, links diagnosis with targeted intervention. Predicted outcomes indicate that implementing this path could raise the course excellent rate to 36.8% by 2028, an improvement of 10 percentage points relative to the conventional improvement scenario, with average D-SRC for core shortcomings reduced by 45.5% and the proportion of students achieving good or higher exceeding 80%, providing a scenario-based reference for evaluating the feasibility of the structured improvement framework. Broader applicability requires further validation.

### Limitations

This study has several limitations that should be acknowledged.

*First, single-course scope.* The proposed framework was validated on a single engineering course, Electric Machinery and Drives (EMD), at one university (West Anhui University). This limits statistical generalizability to other courses, disciplines, or institutions. However, the five-year longitudinal design (2021–2025, n = 882) provides a different form of validation: temporal stability and long-term operational feasibility in a real educational setting. Unlike cross-sectional studies that capture a single snapshot, this longitudinal design demonstrates that the framework remains stable and usable over multiple years with consistent data collection protocols. Future research should replicate this framework across multiple courses and institutions to establish broader generalizability. Any claims regarding transferability should be interpreted as preliminary.

*Second, D-SRC parameter α.* The coefficient α was set to 0.2 as a moderate baseline value based on sensitivity analysis, but the optimal value may vary across different evaluation settings. Future research should further examine adaptive or data-driven parameter selection strategies.

*Third, first-order Markov simplification.* The first-order Markov model simplifies cumulative and context-dependent educational outcomes. Course performance evolution may be affected by curriculum reforms, instructor changes, cohort quality, teaching resources, and policy conditions, which are not fully captured by the current model. The first-order Markov model is used as a simplified approximation of state transitions; however, higher-order temporal dependencies may exist and are not explicitly modeled in this study. Moreover, the five-year observation period limits the robustness of transition-matrix estimation. Therefore, the Markov prediction should be interpreted as a short-term exploratory approximation rather than a robust long-term predictive tool. Future work will address these limitations through multi-institutional validation, longer longitudinal datasets, and more sophisticated modeling approaches.

*Fourth, machine learning baselines.* With only five annual observations, machine learning models would be prone to severe overfitting and would reduce interpretability for teaching practitioners. Therefore, such comparisons were not included as primary baselines in this study. ML-based comparisons are deferred to future research with larger multi-course and multi-institutional longitudinal datasets.

### Implications and future research

The constructed dynamic evaluation–prediction–optimization framework provides an analytical tool with diagnostic usefulness and decision-support value for curriculum reform in engineering education.

- *Course evaluation perspective:* The framework supports a transition from static cross-sectional assessment to dynamic temporal prediction. The sliding-window entropy-weighting method (*T* = 5) may provide a reference for similar rapidly evolving engineering courses, such as smart manufacturing technology and advanced motor control courses. M-TOPSIS accounts for correlations in multi-indicator evaluation systems without overclaiming superiority; the previously reported 10.7% SD-based improvement is interpreted as increased dispersion rather than direct proof of ranking quality.
- *Instructor-level perspective:* The D-SRC coefficient enables targeted resource allocation to indicators most constraining overall performance, supporting more focused resource allocation. For instance, in 2025, 60% of course reform resources were allocated to b15 (Engineering Ethics and Safety Responsibility) based on D-SRC diagnostics. Incorporation of the trend dimension allows instructors to not only address current weaknesses but also identify potential risks proactively. Indicators with subjective components are standardized operational measures rather than fully objective constructs, with structured rubrics, rater briefings, and annual calibration ensuring consistency.
- *Teaching management perspective*: Markov chain prediction combined with Bootstrap confidence intervals provides a data-backed and uncertainty-quantified baseline for short-term planning. For example, the 2026 projected excellent rate of 26.8% provides a planning reference, with the 95% confidence interval [24.1%, 29.5%] providing uncertainty bounds for management decisions. The first-order Markov model simplifies cumulative and context-dependent educational outcomes and should be interpreted as a short-term exploratory approximation rather than a robust long-term predictive tool.
- *Future research*: As noted in “Limitations” section, validating the proposed decision-support framework across additional engineering courses and multiple universities is a priority for future research. Such validation would establish the conditions under which the framework generalizes and where it requires adaptation. Comparative studies with alternative MCDM frameworks and machine learning baselines would also be valuable as larger longitudinal datasets become available. Additional research may explore deep learning integration with Markov models to track individual student trajectories and generate personalized learning path recommendations, as well as statistical inference for D-SRC significance and online course quality assessment. Future research may also explore advanced AI-based decision-support tools when larger longitudinal datasets become available.
- *Frontier technology integration*: Future research may explore advanced AI-based decision-support tools when larger longitudinal datasets become available. Future research may also explore adaptive AI models and systematic monitoring strategies to further enhance the robustness and scalability of the proposed framework.

## Data Availability

The data presented in this study are available on request from the corresponding author. The data are not publicly available due to privacy concerns for the participants involved in the study.
